# Transfer RNA-Derived Fragments and isomiRs Are Novel Components of Chronic TBI-Induced Neuropathology

**DOI:** 10.3390/biomedicines10010136

**Published:** 2022-01-08

**Authors:** Noora Puhakka, Shalini Das Gupta, Niina Vuokila, Asla Pitkänen

**Affiliations:** A.I. Virtanen Institute for Molecular Sciences, University of Eastern Finland, 70211 Kuopio, Finland; shalini.gupta@uef.fi (S.D.G.); niina.vuokila@uef.fi (N.V.); asla.pitkanen@uef.fi (A.P.)

**Keywords:** TBI, tRNA, miRNA, isomiR, neuroinflammation, RNAseq

## Abstract

Neuroinflammation is a secondary injury mechanism that evolves in the brain for months after traumatic brain injury (TBI). We hypothesized that an altered small non-coding RNA (sncRNA) signature plays a key role in modulating post-TBI secondary injury and neuroinflammation. At 3threemonths post-TBI, messenger RNA sequencing (seq) and small RNAseq were performed on samples from the ipsilateral thalamus and perilesional cortex of selected rats with a chronic inflammatory endophenotype, and sham-operated controls. The small RNAseq identified dysregulation of 2 and 19 miRNAs in the thalamus and cortex, respectively. The two candidates from the thalamus and the top ten from the cortex were selected for validation. In the thalamus, miR-146a-5p and miR-155-5p levels were upregulated, and in the cortex, miR-375-3p and miR-211-5p levels were upregulated. Analysis of isomiRs of differentially expressed miRNAs identified 3′ nucleotide additions that were increased after TBI. Surprisingly, we found fragments originating from 16 and 13 tRNAs in the thalamus and cortex, respectively. We further analyzed two upregulated fragments, 3′tRF-IleAAT and 3′tRF-LysTTT. Increased expression of the full miR-146a profile, and 3′tRF-IleAAT and 3′tRF-LysTTT was associated with a worse behavioral outcome in animals with chronic neuroinflammation. Our results highlight the importance of understanding the regulatory roles of as-yet unknown sncRNAs for developing better strategies to treat TBI and neuroinflammation.

## 1. Introduction

Traumatic brain injury (TBI) is caused by an external mechanical force to the head [1]. Epidemiologic studies indicate that TBI is most commonly caused by falls and traffic accidents [2]. Importantly, TBI is the most common cause of disability in people under 40 [1]. After the impact, the primary injury triggers a cascade of molecular dysregulation [3,4], leading to chronic neuroinflammation [5,6]. These changes contribute to the development of secondary injuries and chronic post-TBI comorbidities, such as cognitive impairment [7,8] and epilepsy [9]. Studies of the mechanisms of secondary injury and recovery processes are essential to identify molecular targets for therapeutic interventions as there are currently no medications available that alleviate the post-TBI aftermath. Recent technologic advances toward exploring the transcriptome have led to the discovery of several new classes of small non-coding RNAs (sncRNAs) with potentially key regulatory roles in normal brain function and disease [10,11]. Commonly, sncRNAs are defined as transcripts of <200 nucleotides (nts) that lack a protein-coding capacity [12]. Understanding the functional role of sncRNAs might lead to the development of new strategies to combat complex brain disorders like TBI.

Among sncRNAs, microRNAs (miRNAs) and miRNA-regulated mechanisms are most thoroughly studied in brain diseases [13]. MicroRNAs are small (19–22 nts) RNA molecules that regulate the expression of target genes at the post-transcriptional level via sequence-specific (seed) binding to the 3′ untranslated region [14]. After experimental TBI, dysregulated miRNAs reportedly target such processes as cellular functions, transcription, signal transduction, growth, protein modification, and response to stress, which can further impair recovery [15]. While most studies report dysregulation of canonical miRNA, less emphasis has been paid to alterations in the gene expression of miRNA isoforms, i.e., isomiRs [16,17]. Currently, more attention is being focused on isomiRs and researchers are beginning to unravel the complex functions of these molecules and modification events, which have been long underappreciated.

Another class of sncRNAs, transfer RNAs (tRNAs), is best known for its canonical role in protein translation [18]. Interestingly, various stress conditions can induce tRNA cleavage into short tRNA-derived fragments (tRFs) [19]. This process is potentially mediated by endoribonucleases, such as angiogenin and dicer [20]. The biogenesis details of tRFs, however, are not well known [21]. Increasing evidence reveals that tRFs can modulate diverse biologic processes, including cell proliferation and apoptosis [18,19,22,23,24]. Further, tRFs are reported to display miRNA-like activity, thereby influencing posttranscriptional regulation of their messenger RNA (mRNA) targets [24]. To date, however, few studies have investigated the role of tRFs in brain pathophysiology [10,25,26,27].

In the present study, we performed genome-wide sequencing of sncRNAs and mRNAs of samples originating from post-TBI animals phenotyped to display chronic neuroinflammation. We hypothesized that an altered sncRNA signature plays a key role in modulating post-TBI neuroinflammation and secondary injury. We analyzed canonical and isomiR profiles, and quantified dysregulated tRFs in the perilesional cortex and ipsilateral thalamus at three months post-TBI. We report an association between increased expression of the full miR-146a profile (canonical miRNA and all isomiRs), 3′tRF-IleAAT and 3′tRF-LysTTT, and a worse behavioral outcome in animals with chronic neuroinflammation after TBI. Our results highlight the importance of understanding the regulatory roles of as-yet unknown sncRNAs for the development of better strategies to treat TBI.

## 2. Materials and Methods

### 2.1. Animals

**Figure 1:**
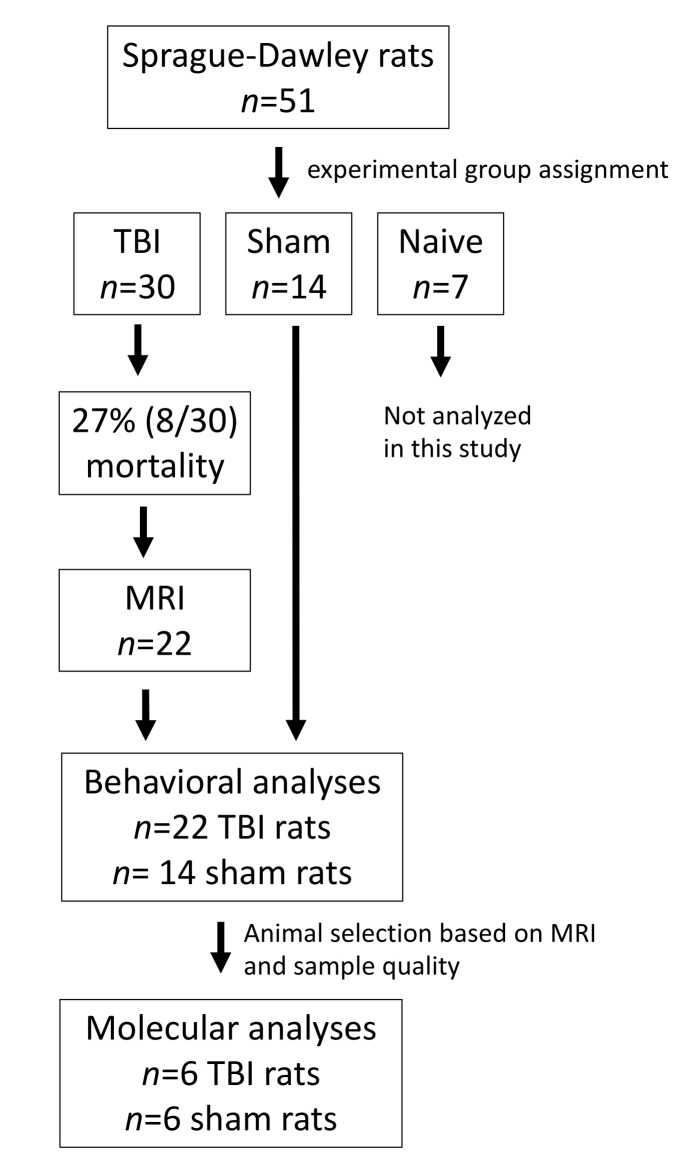
Study design.

A total of 51 adult male Sprague-Dawley rats (mean body weight at the time of injury, 347–425 g; Harlan Laboratories S.r.l., Udine, Italy) were used in this study. The study design is presented in Figure 1. The rats were individually housed in a controlled environment (temperature 22 ± 1 °C; humidity 50–60%; lights on from 07:00–19:00 h). Pellet food and water were provided ad libitum. All animal procedures were approved by the Animal Ethics Committee of the Provincial Government of Southern Finland and performed in accordance with the guidelines of the European Community Council Directives 2010/63/EU.

During the entire study duration, the overall well-being of the rats, as well as their motor activities, eating and drinking behaviors, and tooth growth were observed daily as previously described in detail [28]. If an animal exhibited signs of pain (e.g., weight loss, abnormal movement or posture, excessive grooming), it was treated with carprofen (Rimadyl^®^, 5 mg/kg, once per day for 3 days, Zoetis Finland Oy, Helsinki, Finland). Our pre-study-determined humane endpoint was that if the post-injury weight loss exceeded 30%, the rat would be killed by deep anesthesia induced by 4% isoflurane followed by decapitation. None of the animals in this study met this criterion.

### 2.2. Induction of TBI with Lateral Fluid-Percussion

The procedure for inducing the lateral fluid-percussion injury (FPI) was described previously in detail [29]. Animals (*n* = 30) were anesthetized with an intraperitoneal (i.p.) injection of a solution containing sodium pentobarbital (58 mg/kg), magnesium sulfate (127.2 mg/kg), propylene glycol (42.8%), and absolute ethanol (11.6%), and placed in a Kopf stereotactic frame (David Kopf Instruments, Tujunga, CA, USA). The skull was exposed with a midline skin incision and the periosteum extracted. The left temporal muscle was gently detached from the lateral ridge. A circular craniectomy (Ø 5 mm) was performed over the left parietal lobe midway between the lambda and bregma, leaving the dura mater intact. The edges of the craniectomy were sealed with a modified Luer-lock cap that was filled with saline while the calvaria was covered with dental acrylate (Selectaplus CN, Dentsply DeTrey GmbH, Dreieich, Germany). Lateral FPI was produced 90 min after the induction of anesthesia by connecting the rat to a fluid-percussion device (AmScien Instruments, Richmond, VA, USA) via a female Luer-lock fitting. The mean severity of the impact was 3.3 ± 0.01 atm. Sham-operated control animals (*n* = 14) underwent anesthesia and all surgical procedures, but not the impact. Seven naïve animals were included in the cohort but were not used in the present study.

### 2.3. Composite Neuroscore

To assess somatomotor recovery, the rats (*n* = 22 TBI animals and *n* = 14 shams) underwent composite neuroscore testing at baseline (pre-injury), and at 2, 7, 14, 21, and 90 days post-TBI (for details, see [30]). Animals were scored from 0 (severely impaired) to 4 (normal) in the following categories: (1) left and right forelimb contraflexion while suspended by the tail, (2) left and right hindlimb flexion when gently pulled back by the tail, (3) ability to resist left and right lateral pulsion, and (4) ability to stand on an inclined surface. The first 3 categories allow for a maximum of 4 points from both the left and right sides. The last category was scored from 0 to 4, leading to a maximum total score of 28 points.

### 2.4. Magnetic Resonance Imaging

To distinguish rats with a chronic perilesional inflammatory rim, T2-weighted magnetic resonance imaging (MRI) was performed at 2 months post-TBI (*n* = 22 TBI animals) using a 7 T Bruker Pharmascan MRI scanner equipped with a volume transmitter coil and surface receiver coil combo [28]. Briefly, the rats were anesthetized with isoflurane (4% for induction and 2% for maintenance) and positioned in a stereotactic holder. Thereafter, T2-weighted images were acquired with fast spin-echo from 25 slices (field of view = 30 mm × 30 mm, matrix slice thickness = 1 mm, TR = 4000 ms, TE = 40 ms).

### 2.5. Morris Water Maze

The spatial learning and memory performance of the rats (*n* = 22 TBI animals and *n*= 14 shams) was tested in a Morris water maze using a 3-day paradigm [31]. At the end of day 3, the platform was removed from the maze apparatus and rats were allowed to swim for 60 s to evaluate their memory of the platform location (probe trial). The time spent in each of the 4 quadrants of the maze was recorded.

### 2.6. Sampling of Brain Tissue

Rats (*n* = 22 TBI animals and *n* = 14 shams) were anesthetized with 5% isoflurane and decapitated, and their brains were quickly removed. Each brain was flushed with 0.9% cold (4 °C) sodium chloride to remove blood and hair and placed onto a slicing matrix on ice (#15007, Rodent Brain Matrix, Ted Pella, Inc., Redding, CA, USA). One 3-mm-thick coronal slice was cut (between −1 and −4 from the bregma), from which the perilesional cortex and ipsilateral thalamus were dissected on top of the glass plate placed on ice. Brain tissue samples were snap-frozen in liquid nitrogen and stored at −70 °C. All tissue preparations were performed in under 12 min (range 9–12 min) after decapitation. The remaining brain tissue was placed in a 10% formalin solution for 3 days, followed by cryoprotection in 20% glycerol in a 0.02 M PB buffer for 2 days. Brain pieces were snap-frozen on dry ice and stored at −70 °C until further processed.

### 2.7. Histologic Analysis

For histologic analysis, brains pieces were sectioned (1-in-6 series, 30 µm) with a sliding microtome (Leica SM 2000, Leica Microsystems Nussloch GmbH, Nussloch, Germany). The sections were stored in a cryoprotectant solution (30% ethylene glycol, 25% glycerol in 0.05 M sodium phosphate buffer, pH 7.4) until stained.

Nissl staining. To further characterize the lesion pathology (*n* = 22 TBI animals and *n*= 14 shams), we performed Nissl staining (see [32]).

Immunohistochemical staining for CD68 and GFAP. Freely floating sections were transferred to 24-well nets for staining. The complete staining protocol for astrocyte (glial fibrillary acidic protein [GFAP]) and microglial/macrophage response (CD68) immunohistochemistry (*n* = 5 representative post-TBI animals), was previously described in detail by Huusko et al. [32] with slight modifications. As a primary antibody, we used a mouse monoclonal antibody to GFAP (1:2000, #814369, Boehringer, Mannheim, Germany), and a mouse monoclonal antibody to rat CD68 (1:1000, EMD Millipore, Billerica, MD, USA). One representative brain section per rat was selected using the MRI images as a guide for determining the lesion location and extent. For GFAP, sections were incubated in the primary antibody mixture for 2 days. For CD68, the sections were treated for 15 min with 1% sodium borohydride for antigen retrieval prior to incubation in a blocking solution. In addition, 0.1% Triton-X 100 was used in all steps. Similar to GFAP, the sections were incubated in primary antibody for 2 days.

### 2.8. Isolation of Total RNA from Brain Tissue

RNA was extracted from the perilesional cortex and ipsilateral thalamus (*n* = 6 TBI animals and *n* = 6 shams) using a mirVana miRNA isolation kit (#AM1560, Life Technologies (Ambion) Carlsbad, CA, USA), QIAshredder (#79654, Qiagen, Hilden, Germany), and AllPrep DNA/RNA Mini Kit (#80204, Qiagen) as previously described in detail [33,34]. Briefly, to avoid clogging the spin columns, brain tissue was divided into 2–4 pieces (max ~10 mg each) on dry ice. Each tissue piece was then placed into a 2-mL microcentrifuge tube together with 1 metal ball and 800 μL of Ambion Lysis/binding buffer and homogenized with a TissueLyser (Qiagen) for 3 min (30 Hz). For further homogenization, the lysate was transferred to a QIAshredder spin column and centrifuged (16,000× *g*) for 2 min at 4 °C. Flow-through lysate was transferred back to the QIAshredder spin column and centrifuged a second time. For DNA separation, tissue lysate was transferred to a Qiagen All Prep DNA spin column and centrifuged (10,000× *g*) for 1 min at room temperature. Flow-through from the All Prep DNA spin column was used for RNA extraction using a mirVana miRNA isolation kit. Briefly, miRNA homogenate additive (70 μL) was added to the flow-through. The mixture was vigorously vortexed for 30 s and then incubated on ice for 10 min. Acid-phenol:chloroform (700 μL) was then added, mixed, and centrifuged (16,000× *g*) for 30 s. The aqueous upper phase was transferred to a new microcentrifuge tube. A total of 500 µL of water was added to the lower phase, which was then mixed and centrifuged (16,000× *g*) for 30 s. The upper aqueous phase was collected into the same tube as the aqueous phase from the previous extraction cycle. Then, 100% ethanol (625 μL) was added to the tube, mixed, and transferred to the mirVana miRNA isolation spin column. Finally, RNA was washed and eluted from the spin column according to instructions provided with the mirVana miRNA isolation kit. Finally, RNA extracted from each brain region was pooled. Thereafter, RNA purification was performed using a miRCURY™ RNA Isolation Kit (#300111, Exiqon, Vedbaek, Denmark) according to the manufacturer’s instructions.

The concentrations and 260/280 ratios of the eluted RNA from the perilesional cortex and ipsilateral thalamus were measured using a NanoDrop 1000 spectrophotometer. RNA quality and the RNA integrity number were measured with an Agilent 6000 Nano kit (#5067–1511, Agilent Technologies, Waldbronn, Germany) using an Agilent Bioanalyzer. The RNA integrity numbers (range 8.9–9.2) and 260/280 ratios (range 2.01–2.10) prior to the sequencing service and for samples in the later polymerase chain reaction (PCR) study are shown in Supplementary Table S1.

### 2.9. Small RNA and RNA Sequencing from Brain Tissue

All sequencing experiments and data analysis (*n* = 6 TBI animals and *n* = 6 shams) were conducted at Exiqon Services, Denmark.

#### 2.9.1. Small RNA Sequencing

Library preparation and next-generation sequencing. First, 300 ng of total RNA was converted to microRNA NGS libraries using a NEBNEXT library generation kit (New England Biolabs Inc., Ipswich, MA, USA) according to the manufacturer’s instructions. Each individual RNA sample then had adaptors ligated to its 3′ and 5′ ends and converted into cDNA. The cDNA was pre-amplified with specific primers containing sample-specific indexes. After 15 cycles pre-PCR, the libraries were purified on QiaQuick columns, and the insert efficiency was evaluated by a Bioanalyzer 2100 instrument on a high sensitivity DNA chip (Agilent Inc., Santa Clara, CA, USA). The microRNA cDNA libraries were size-fractionated on a LabChip XT (Caliper Inc., Princeton, NJ, USA) and a band representing the adaptors and a 15–40 bp insert was excised according to the manufacturer’s instructions. Samples were then quantified by quantitative PCR (qPCR) and concentration standards. Based on the quality of the inserts and the concentration measurements, the libraries were pooled in equimolar concentrations (all concentrations of libraries to be pooled were of the same concentration). The library pool(s) were finally quantified again with qPCR and the optimal concentration of the library pool used to generate the clusters on the surface of a flow cell before sequencing with a v3 sequencing methodology according to the manufacturer’ instructions (Illumina Inc., San Diego, CA, USA). Finally, the samples were sequenced on the Illumina NextSeq 500 system. The system uses quality score binning, which enables a more compact storage of raw sequences. The method was tested using only 8 levels (Levels: No call, 6, 15, 22, 27, 33, 37, and 40) of quality and determined to be virtually loss-less (http://res.illumina.com/documents/products/whitepapers/whitepaper_datacompression.pdf; accessed on 16 June 2015).

Data analysis. After the sequencing, intensity correction, base calling, and assigning of Q-scores was performed. Adapters were trimmed off as part of the base calling. Subsequently, the data were quality checked with FastQC [35]. Sequences were mapped against rat reference genome RGSC3.4 and annotated with mirBase 20. Differential expression analyses were performed using the edgeR statistical software package (Bioconductor, http://www.bioconductor.org/; accessed on 10 December 2021). For normalization, the trimmed mean M-value method was used based on log-fold and absolute gene-wise changes in expression levels between samples (TMM normalization).

Identification of isomiRs. IsomiR analysis was performed individually for each sample based on the occurrence of count variants for each detected microRNA. Reads were mapped to known miRNAs according to the annotation in the miRBase release 20 and then investigated for the presence of different isomiRs. These variants were identified by changes in the start or stop position, or the occurrence of mutations within the read. The results for each sample were then merged to generate a single count file with a consistent nomenclature across the samples. Only isomiRs that were present at a level of 5% of the total reads for that miRNA were retained.

Identification of tRNA derived fragments. Reads mapped against small RNA (including tRNA) in the rat reference genome RGSC3.4 were counted and visualized with an Integrative Genomics Visualizer (IGV; [36]). Differential gene expression analysis was performed similarly as for miRNAs to assess overall change in tRFs originating from one particular tRNA. Two candidate tRFs were selected for further experiments as they had a clear cleavage site based on visual analysis with the IGV.

#### 2.9.2. Messenger RNA Sequencing

Library preparation and next-generation sequencing. The library was prepared using a TruSeq^®^ Stranded mRNA Sample preparation kit (Illumina inc.). The starting material (300 ng) of total RNA was mRNA enriched using the oligo (dT) bead system. The isolated mRNA was subsequently fragmented by enzymatic fragmentation. Then, first-strand and second-strand syntheses were performed and the double-stranded cDNA was purified (AMPure XP, Beckman Coulter, Brea, CA, USA). The cDNA was end-repaired and 3′ adenylated, Illumina sequencing adaptors were ligated onto the fragments ends, and the library was purified (AMPure XP). The mRNA stranded libraries were pre-amplified with PCR and purified (AMPure XP). The libraries’ size distribution was validated and quality inspected on a Bioanalyzer high sensitivity DNA chip (Agilent Technologies). High quality libraries were quantified by qPCR, the concentration normalized, and the samples pooled according to the project specification (number of reads). The library pool(s) were re-quantified with qPCR and the optimal concentration of the library pool was used to generate clusters on the surface of a flow cell before sequencing on the Nextseq500 instrument using a High Output sequencing kit (75 cycles) according to the manufacturer’s instructions (Illumina Inc.).

Data analysis. TopHat (v2.0.11) was used to align the sequencing reads to the reference genome (RGSC3.4) with the sequence aligner Bowtie2 (v.2.2.2). Cufflinks (v2.2.1) was used to take the alignment results from TopHat and assemble the aligned sequences into transcripts, thereby constructing a map or a snapshot of the transcriptome. Reference genome version RGSC3.4 was used. To guide the assembly process, an existing transcript annotation (Rnor_5.0 Ensembl) was used. When comparing groups, Cuffdiff [37] was used to calculate the FPKM (number of fragments per kilobase per million mapped fragments) and test for differential expression and regulation among the assembled transcripts across the submitted samples using the Cufflinks output.

### 2.10. Pathway Analysis of RNA-Seq Data

Qiagen Ingenuity pathway analysis (IPA^®^, Qiagen; Content version 31813283) was used to assess regulated molecular pathways and gene networks. IPA analysis was performed using “Experimentally Observed” and “High (predicted)” confidence settings. The cutoff was set to FDR <0.05 (both up/downregulated). After filtering, the dataset from the perilesional cortex included 4653 genes. Similarly, 5256 genes from the ipsilateral thalamus were included in the analysis.

### 2.11. Validation of the Small RNA-Seq Data with Droplet Digital PCR and Quantitative PCR

#### 2.11.1. microRNA

Reverse transcription (RT) of total RNA from brain tissue with TaqMan chemistry (*n* = 6 TBI animals and *n* = 6 shams). Validation of the small RNA-Seq data was performed from the same samples subjected to sequencing (*n* = 6 TBI and 6 sham-operated controls). All RNA samples were diluted 1:100 with RNAase-free water prior to cDNA synthesis. In the perilesional cortex, validation was performed for the following miRNAs: miR-132-5p (002132), miR-212-5p (463930_mat), miR-375-3p (000564), miR-20a-5p (00580), miR-211-5p (001199), miR-135a-3p (002075), miR-339-5p (002257), miR-34c-3p (000426), miR-128-3p (002216) and miR-17-5p (002308). In the ipsilateral thalamus, validation was performed for miR-146a-5p (002163) and miR-155-5p (002571). For normalization, 4 miRNAs were selected from the small RNA-Seq data as endogenous controls: miR-378a-3p (002243), miR-3594-3p (464929_mat), miR-330-5p (002230), and let-7b-3p (002404). Reverse transcription for all target and control miRNAs was performed with the TaqMan protocol (TaqMan miRNA Reverse Transcriptase Kit #4366596, Applied Biosystems, Foster City, CA, USA https://www.thermofisher.com/document-connect/document-connect.html?url=https%3A%2F%2Fassets.thermofisher.com%2FTFS-Assets%2FLSG%2Fmanuals%2F4364031_TaqSmallRNA_UG.pdf; accessed on 10 December 2021), according to the manufacturer’s instructions. Samples were stored at −20 °C until further processed.

Droplet digital PCR (*n* = 6 TBI animals and *n* = 6 shams). Absolute quantification of miRNA copy numbers was performed with droplet digital PCR (ddPCR). For the perilesional cortex, cDNA from miR-378a-3p was diluted 1:6, and miR-128-3p was diluted 1:8 in ddPCR. All other cDNA samples were used undiluted. For the ipsilateral thalamus samples, cDNA from miR-378a-3p and miR-330-5p were diluted 1:4 in ddPCR. Undiluted cDNA was used for all other miRNAs. The cDNA template (1.33 μL) was added to a 20-μL reaction mixture containing 10 μL Bio-Rad 2x ddPCR supermix for probes (#186-3010, Bio-Rad, Hercules, CA USA), 1 μL 20× miRNA PCR primer, and 7.67 μL nuclease-free water. The 20-μL reaction mixtures were loaded in disposable droplet generator cartridges (#186-4008, DG8TM Cartridges for QX100TM/QX200TM Droplet Generator, Bio-Rad, Germany) along with 70 μL of droplet generation oil for probes (#186-3005, Bio-Rad, CA, USA). The cartridges were covered with gaskets (#186-3009, Droplet Generator DG8TM Gaskets, Bio-Rad, Hercules, CA, USA) and placed in the droplet generator (#186-3001, Bio-Rad QX100TM Droplet Generator, Solna, Sweden) for creating the oil-emulsion droplets. The generated droplets were transferred to 96-well PCR plates (Twin. tec PCR plate 96, semi-skirted, colorless, Hamburg, Germany) sealed, thermal-cycled (96-well PTC-200 thermal cycler, MJ Research), and quantified at the end-point in the droplet reader (#186-3001, Bio-Rad QX100TM Droplet Reader, Solna, Sweden) using QuantaSoft software (v1.7, Product code: 186-4011, Bio-Rad, USA). For each sample, the reaction was performed in duplicate, and the average miRNA copies/20 μL well from the 2 replicates was calculated.

To normalize PCR measurements, the total RNA input to the cDNA reaction was first calculated based on the NanoDrop concentrations and the implemented dilution factor (1:100). For each miRNA, the average number of copies/20 μL well was then normalized to the total RNA input, to account for the slight sample-to-sample variations in the RNA input volumes. Normalization was performed as follows:

The miRNA copies normalized to RNA input = (average miRNA copies/20 μL well)/(total RNA input to the 15 μL cDNA reaction)

Next, the geometric mean (GM) of the RNA input-normalized copies from the 4 endogenous controls, miR-378a-3p, miR-3594-3p, miR-330-5p, and let-7b-3p, was calculated. Finally, the target miRNAs were normalized to the geometric mean of the 4 endogenous controls as follows:

Normalized target miRNA copies = (miRNA copies normalized to RNA input)/(GM of RNA input normalized miRNA copies from 4 endogenous controls)

IsomiR analysis of selected candidate miRNAs. For all candidate and control miRNAs tested for validation with ddPCR, the non-normalized isomiR read count list was obtained for each biologic sample from the small RNA-Seq data. For each miRNA, the total read count from all isomiRs, and the number of detected isomiRs were compared among the TBI and sham-operated controls. Next, analysis was focused on the miRNA candidates that were validated with ddPCR: miR-375-3p, miR-211-5p, and miR-146a-5p. We excluded miR-155-5p because it had a low read abundance in all samples, with no detected isomiRs. For miR-375-3p, miR-211-5p, and miR-146a-5p, the top 6 isomiRs (based on read count abundance) from all TBI samples were listed, and among them, the top 2–4 isomiRs that were detected in all the TBI samples were considered. For each of these 2–4 isomiRs, the read count, change involved (if any), percentage of total reads comprising the isomiR, and percentage of canonical sequence reads comprising the isomiR were tabulated and compared between the TBI and sham-operated controls.

Reverse transcription of total RNA from brain tissue with miScript chemistry. Validation of the small RNA-Seq data was performed from the same samples subjected to sequencing (*n* = 6 TBI animals and 6 sham-operated controls). Synthesis of cDNA was performed with miScript II RT kit (#218161, Qiagen) according to the manufacturer’s instructions.

Quantitative PCR analysis of miR-146a with miScript chemistry. Quantitative RT-PCR analysis was performed using miScript chemistry according to the manufacturer’s instructions. For this, a miScript SYBR Green PCR Kit (#218073, Qiagen) was used with a rno-miR-146a specific 5′ end primer (MS00000441, Qiagen) and a universal 3′ end primer. For normalization, we used miR-378a-3p (MS00005810, Qiagen) with the formula 2-ΔCt [38]. For each sample, the PCR reactions were performed in triplicate. All samples were run with a LightCycler^®^ 96 Instrument (Roche). A no-template (nuclease-free water) control was run in parallel for all assays.

#### 2.11.2. Transfer RNA Derived Fragments

Reverse transcription of total RNA from brain tissue with miScript chemistry (*n* = 6 TBI animals and *n* = 6 shams). Validation of the small RNA-Seq data was performed from the same samples subjected to sequencing (*n* = 6 TBI and 6 sham-operated controls) as described in the miRNA paragraph for miScript chemistry.

Quantitative PCR (*n* = 6 TBI animals and *n* = 6 shams). Relative quantification of tRNA fragments was performed as described for miR-146a with miScript chemistry. Custom designed assays for tRFs derived from the 3′ end of the tRNA-IleAAT (MSC0076231, Qiagen) and tRNA-LysTTT (MSC0076337, Qiagen) were used.

Agarose gel electrophoresis for size separation (*n* = 3 TBI animals and *n* = 3 shams). First, 2 μL of RNA (150 ng), 16 μL of formamide, and 2 μL of loading dye were mixed and then heated at 65 °C for 5 min. For size separation of the small RNA, 3% agarose gel (#A9539-500G, Sigma, Tokyo, Japan) was prepared in a 1 × Tris-acetate buffer (TAE) and run 15 min with 80V in 1 × TAE. A GeneRuler low range DNA ladder (#SM1193, Thermo Scientific, Waltham, MA, USA) and Sybr Safe DNA gel stain (#S33102, Invitrogen, Waltham, MA, USA) were used to visualize the RNA size. An appropriate piece of the gel was cut and minced with a microtome blade. Small RNA was eluted from the gel to nuclease-free water (600 μL) at room temperature on a shaker overnight. Complementary cDNA and reverse transcription (RT)-qPCR was performed as described for the miScript chemistry earlier, except data normalization was performed relative to miR-124-3p (MS00005593, Qiagen). Undiluted RNA after elution was directly used for the cDNA reaction.

### 2.12. In-Silico Prediction and qPCR Validation of mRNA Targets for the Validated miRNAs

In-silico prediction. For the validated miRNA candidates, mRNA target prediction was performed in-silico with TargetScan v7.2. The predicted target list was then compared with the differentially expressed mRNA data (FDR <0.05) obtained from our brain RNA-Seq samples. Only those targets that were predicted in TargetScan and present in our differentially expressed mRNA list with a fold-change (FC) opposite that of the miRNA expression were selected for DAVID gene-Gene Ontology term enrichment analysis. Based on prior literature, evidence of miRNA-mRNA interaction, magnitude of fold-change, and read abundance identified from RNA-Seq; 2 mRNA targets were selected for validation with RT-qPCR, ELAVL2 from the perilesional cortex (predicted target of both miR-375-3p and miR-211-5p) and Syt1 from the ipsilateral thalamus (predicted target of miR-146a-5p).

Complementary DNA (cDNA) synthesis (*n* = 6 TBI animals and *n* = 6 shams). The cDNA synthesis was performed using the High Capacity RNA-to-cDNA Kit (Applied Biosystems) according to the manufacturer’s instructions (High Capacity RNA-to-cDNA kit Protocol, Part Number 4,387,951 Rev. C). The total amount of RNA was set to 2 μg/20 μL reaction.

Quantitative RT-PCR (*n* = 6 TBI animals and *n* = 6 shams). Quantitative RT-PCR was performed in a total volume of 20 μL using 12 ng (RNA equivalents) of cDNA as a template, gene-specific primers and probes (pre-validated TaqMan Gene Expression Assay for Elavl1 and Syt1; Rn01433257_m1 and Rn00436862, Applied Biosystems), and 1× TaqMan Gene Expression Master Mix (#4369016, Applied Biosystems, Waltham, MA, USA). The following program was used in the PCR (StepOne Software v2.1, Applied Biosystems): 1 cycle (95 C, 10 min) and 40 cycles (95 C, 15 s; 60 C, 60 s) in a StepOnePlusTM Real-Time PCR System (Applied Biosystems). The data were normalized to glyceraldehyde 3-phosphate dehydrogenase (Gapdh) mRNA expression (pre-validated Taqman Gene Expression Assay for Gapdh; ID: Rn99999916_s1, Applied Biosystems). Each sample was run in triplicate. A no-template (nuclease-free water) control was run in parallel for all assays.

### 2.13. In-Silico Prediction and qPCR Vvalidation of mRNA Targets for the Validated tRFs

In-silico prediction. To predict miRNA-like regulation of tRFs, TargetRank [39] was used to find complementary regions from mRNA 3′ untranslated regions (3′UTRs). Seed regions (8-nt long) were formed along the length of the tRF. For gene set enrichment analysis of the predicted targets for the first 8-nt seed region, a gene list of targets for both tRNA-IleAAT and tRNA-LysTTT was created based on a human database (Supplementary Material 2). Thereafter, the acquired gene list was transformed to official rat gene symbols (334 genes). A pre-ranked gene list was created according to the RNA-Seq dataset. The genes were arranged into negative and positive groups according to the fold-change values. Downregulated genes were identified with a minus sign. The genes were then arranged according to their p-value and ranked. Genes with high p-values were ranked closer to zero. The pre-ranked gene list created for the gene enrichment analysis comprised 18893 features (genes). FDR <0.05 was considered statistically significant. After gene set enrichment analysis, all negatively enriched targets (Supplementary Material 2) were included for further Functional Annotation Tool analysis (DAVID Bioinformatics Resources 6.8, NIAID/NIH). A Benjamini–Hochberg-corrected *p*-value <0.05 was used to find the regulated functions. One candidate target (Cplx1) was selected for further proof-of-concept validation.

Complementary DNA (cDNA) synthesis (*n* = 5 TBI animals and *n* = 5 shams). The cDNA synthesis was performed as described for the predicted miRNA targets.

Quantitative RT-PCR (*n* = 5 TBI animals and *n* = 5 shams). Quantitative RT-PCR was performed as described for the predicted miRNA targets with gene-specific primers and probes (prevalidated TaqMan Gene Expression Assay for Cplx1; Rn02396766_m1, Applied Biosystems).

### 2.14. qPCR Analysis of tRF Cleaving Enzyme Angiogenin

Complementary DNA (cDNA) synthesis (*n* = 5 TBI animals and *n* = 5 shams). The cDNA synthesis was performed as described for the predicted miRNA targets.

Quantitative RT-PCR (*n* = 5 TBI animals and *n* = 5 shams). Quantitative RT-PCR was performed as described for the predicted miRNA targets with gene-specific primers and probes (prevalidated TaqMan Gene Expression Assay for angiogenin; Rn03416813_gH, Applied Biosystems).

### 2.15. Data Analysis and Statistics

No statistical methods were used to predetermine the sample size. Unsupervised hierarchical clustering (R environment) was used to cluster animals and visualize differentially expressed miRNAs and tRFs. The Vienna RNAfold web server (http://rna.tbi.univie.ac.at/cgi-bin/RNAWebSuite/RNAfold.cgi, accessed on 16 June 2015) was used to predict the RNA folding. In RT-qPCR analyses, statistical differences were estimated using the Mann–Whitney U-test (Prism 9). Differences in the neuroscore between 2 groups were assessed using the Mann–Whitney U-test (Prism 9). All correlations were analyzed with the Spearman rank correlation (Prism 9). A *p* < 0.05 was considered to indicate statistical significance. We did not exclude any data points in this study.

## 3. Results

### 3.1. Mortality after Lateral FPI

Acute post-TBI mortality was 27% (8/30). The mean post-impact apnea time was 38 ± 3.6 s. Post-impact seizure-like behaviors were observed in 30% (9/30) of the TBI animals.

### 3.2. Anatomic Analysis and Assessment of Chronic Neuroinflammation

**Figure 2:**
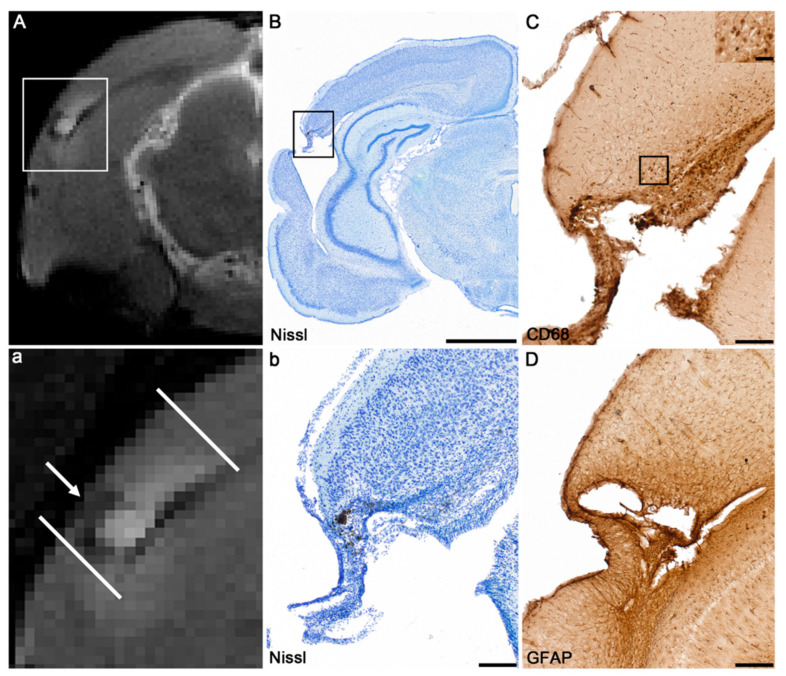
Assessment of chronic neuroinflammation. (**A**) Magnetic resonance imaging (MRI) was used to identify animals with a prominent increase in T2-enhancement around the lesion (arrow in a high magnification representation (**a**)) indicating perilesional inflammation. (**B**) Nissl-stained sections show detailed cytoarchitectonics of the perilesional area. (**C**,**D**) Immunohistochemical stainings were used to confirm chronic neuroinflammation suggested by T2-enhancement. Immunohistochemical staining for CD68 (macrosialin) showed activated microglia/macrophages in the perilesional cortex (panel (**C**)). Immunohistochemical staining for glial fibrillary acidic protein (GFAP) with qualitative analysis showed astrocytic fibers (panel (**D**)). Scale bars = 2000 µm (**B**), 200 µm (**b**,**C**,**D**) and 50 µm (insert in panel (**C**)).

Visual analysis of 1-mm-thick coronal MRI slices revealed substantial variability in the extent of the cortical lesion between the animals (example in the Figure 2A). Moreover, the enhanced T2-weighted signal appeared to be unevenly distributed along the rostrocaudal extent of the cortical lesion. MRI was used to identify animals with a prominent increase in T2-enhancement around the lesion (arrow in Figure 2A), indicating perilesional inflammation (*n* = 13/22). Nissl-stained sections (Figure 2B) showed detailed cytoarchitectonics of the perilesional area. Next, immunohistochemical staining was used to confirm chronic neuroinflammation suggested by the T2-enhancement. Immunohistochemical staining for CD68 with qualitative analysis showed activated microglia/macrophages in the perilesional cortex (Figure 2C). Further, immunohistochemical staining for GFAP with qualitative analysis showed astrocytic fibers (Figure 2D).

**Figure 3:**
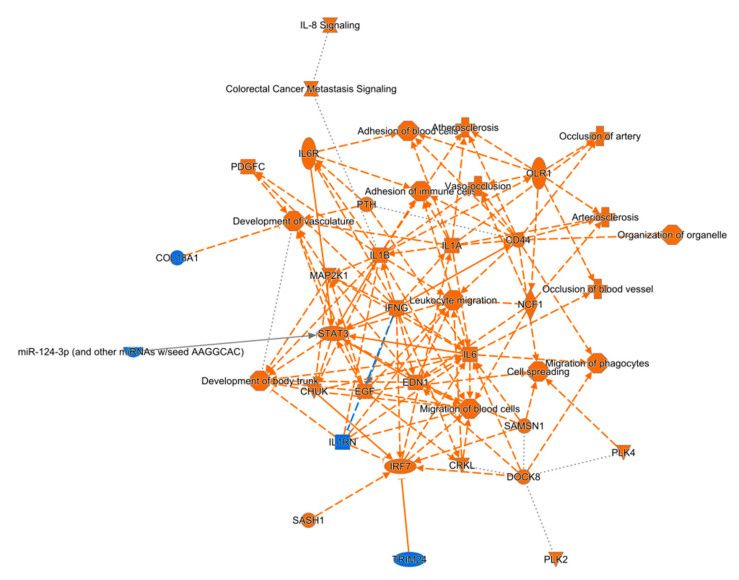
Graphical summary of pathway analysis of differentially expressed genes in the perilesional cortex after traumatic brain injury. Analysis highlighted physiologic functions like organismal survival, tissue morphology, nervous system development and function, and hematologic system development and function.

**Figure 4:**
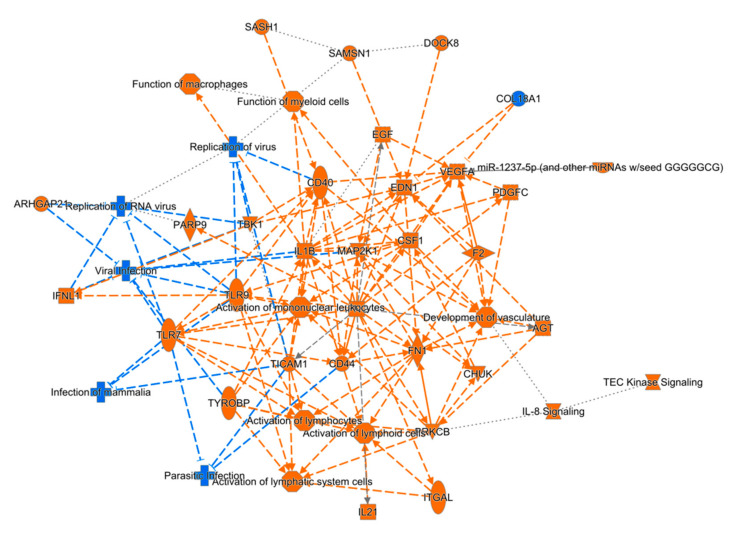
Graphical summary of the pathway analysis of differentially expressed genes in the ipsilateral thalamus after traumatic brain injury. Similar to the perilesional cortex, analysis highlighted physiologic functions, such as organismal survival, tissue morphology, nervous system development and function, and hematologic system development and function. Top regulator effect networks included genes such as Collagen Type XVIII Alpha 1 Chain (COL18A1) and SELPG protein.

RNA-sequencing was used to assess detailed molecular network alterations after TBI in the perilesional cortex (Figure 3) and ipsilateral thalamus (Figure 4). Basic quality control of RNAseq data suggested experimental success (Supplementary Table S2). Overall, we detected 2773 and 2954 upregulated genes in the perilesional cortex and ipsilateral thalamus, respectively (Supplementary Figure S1). The number of downregulated genes was 2490 in the perilesional cortex and 3044 in the ipsilateral thalamus (Supplementary Figure S1).

Top regulator effect networks included genes like CRK Like Proto-Oncogene Adaptor Protein (CRKL), Polo Like Kinase 2 (PLK2), and Neutrophil Cytosolic Factor 1 (NCF1). In the perilesional cortex, the top five regulated canonical pathways were axonal guidance signaling (38% overlap with dataset, 172/456, *p* < 0.001), colorectal cancer metastasis signaling (42% overlap, 106/252, *p* < 0.001), molecular mechanisms of cancer (37% overlap, 142/382, *p* < 0.001), role of nuclear factor of activated T cells in cardiac hypertrophy (44% overlap, 87/199, *p* < 0.001), and role of macrophages, fibroblasts, and endothelial cells in rheumatoid arthritis (38% overlap, 120/315, *p* < 0.001). Similarly, in the ipsilateral thalamus, the top five regulated canonical pathways were interleukin (IL)-8 signaling (51% 100/197, *p* < 0.001), leucocyte extravasation signaling (49% overlap, 103/210, *p* < 0.001), role of nuclear factor of activated T cells in cardiac hypertrophy (50% overlap, 96/192, *p* < 0.001), G-protein coupled receptor signaling (45% overlap, 123/272, *p* < 0.001), and Huntington’s disease signaling (46% overlap, 111/241, *p* < 0.001).

In the perilesional cortex, IPA analysis revealed the top five molecular and cellular functions among differentially expressed genes to be cellular assembly and organization (927 molecules), cellular function and maintenance (1484 molecules), cellular movement (1079 molecules), cellular morphology (1178 molecules), and cellular growth and proliferation (1744 molecules). The top five molecular and cellular functions in the ipsilateral thalamus were cellular assembly and organization (1048 molecules), cellular function and maintenance (1675 molecules), cellular morphology (1321 molecules), molecular transport (1153 molecules), and cell death and survival (1657 molecules).

Based on these data, we also predicted the top five upstream regulators with IPA. In the perilesional cortex, all five of these regulators were activated (*p* < 0.001). These five regulators were TGFB1, tNF, TP53, IFNG, and APP. In the ipsilateral thalamus, of the top five regulators, four (APP, TNF, IFNG, and lipopolysaccharide) were activated and one (L-dopa) was inhibited (*p* < 0.001).

### 3.3. Differentially Expressed miRNAs Identified from sncRNA-Seq

**Figure 5:**
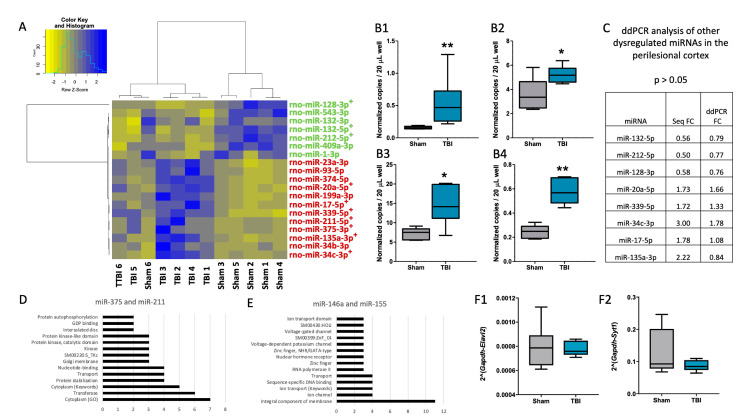
Deregulated microRNAs (miRNAs) after lateral fluid percussion injury. (**A**) Unsupervised hierarchical clustering partly separated sham-operated controls from post-TBI rats according to differentially expressed (**D**,**E**) miRNAs in the perilesional cortex. A total of 12 upregulated and 7 downregulated miRNAs were found (FDR <0.05). (**B1–B4**) Droplet digital PCR (ddPCR) analysis validated miR-375 (**B1**, *p* < 0.01) and miR-211 (**B2**, *p* < 0.05) upregulation. In the ipsilateral thalamus, only 2 miRNAs were upregulated in small non-coding RNAseq analysis (sncRNAseq, FDR <0.05). Both of these were validated with ddPCR (**B3**, miR-146a, *p* < 0.05; **B4**, miR-155, *p* < 0.01). (**C**) All other sncRNAseq suggested no change in the top 10 DE miRNAs in ddPCR analysis (*p* > 0.05 for all). Most of them (7/8), however, were regulated in the same direction detected in the sncRNAseq analysis. (**D**) Functional pathway analysis of predicted targets of miR-375 and miR-211. (**E**) Functional pathway analysis of predicted targets of miR-146a and miR-155. (**F1**,**F2**) Quantitative RT-PCR analysis revealed no change in the predicted targets selected for validation (ELAV Like RNA Binding Protein 2 (Elavl2) and Synaptotagmin 1 (Syt1), *p* > 0.05). Statistical significance: *, *p* < 0.05; **, *p* < 0.01.

Overall, quality control analysis of the sncRNAseq suggested good quality (Supplementary Figure S2A–C). In the perilesional cortex, 19 miRNAs were identified to be differentially expressed in TBI animals at 3 months post-injury compared with controls (FDR <0.05, Figure 5A). In the ipsilateral thalamus, only two miRNAs were identified to be differentially expressed with FDR <0.05. From the 19 differentially expressed miRNAs in the perilesional cortex, PCR analysis was performed for the top 10 candidates (ranked based on increasing order of FDR-value; Figure 5B1,B2,C). In the ipsilateral thalamus, the two differentially expressed miRNAs identified from small RNA-Seq were selected for further validation. Among the 10 miRNA candidates tested from the perilesional cortex with ddPCR, upregulation of only miR-375-3p (FC = 3.44, *p* < 0.01, Figure 5B1) and miR-211-5p (FC = 1.45, *p* < 0.05, Figure 5B2) was validated. For the majority (seven of eight) of the remaining candidates, ddPCR showed a similar expression pattern to that observed with small RNA-Seq (Figure 5C). The PCR analysis results, however, were not statistically significant (*p* > 0.05). From the ipsilateral thalamus, both tested candidates, miR-146a-5p (FC = 2.01, *p* < 0.05, Figure 5B3) and miR-155-5p (FC = 2.34, *p* < 0.01, Figure 5B4), were successfully validated with ddPCR. Similar to the perilesional cortex, these two miRNAs were significantly upregulated in the ipsilateral thalamus of the TBI group at 3 months post-injury compared with sham-operated controls.

### 3.4. mRNA Targets Predicted for the Validated miRNA Candidates

For miR-375-3p, 12/178 (6.7%) of the predicted mRNA targets from TargetScan were also significantly downregulated in our mRNA-Seq data in TBI animals compared with sham-operated controls. For miR-211-5p, 7/98 (7.1%) of the predicted mRNA targets from TargetScan were significantly downregulated in our mRNA-Seq from TBI animals compared with sham-operated controls. The most common functions among these targets were cytoplasmic functions, and transferase and protein stabilization (Figure 5D). Similarly, for miR-146a-5p, 7/137 (5.1%) of the predicted mRNA targets from TargetScan, and for miR-155-5p, 23/288 (7.9%) of the predicted mRNA targets from TargetScan were significantly downregulated in our mRNA-Seq from TBI animals compared with sham-operated controls. The most common functions among these targets were the integral components of membranes, ion channels, and ion transport (Figure 5E). For both miRNAs from the perilesional cortex (miR-375-3p and miR-211-5p), ELAV-like neuron-specific RNA binding protein 2 (ELAVL2) was a common predicted mRNA target. Previous studies revealed a role of miR-375 in regulating dendrite formation and maintenance by affecting the levels of ELAVL4 protein, a paralog of ELAVL2. Thus, ELAVL2 (mRNA-Seq FC = 0.75, FDR <0.05) was selected for validation with RT-qPCR. From the ipsilateral thalamus, synaptotagmin 1 (Syt1) (mRNA-Seq FC = 0.73, FDR <0.05), a predicted target for miR-146a-5p, was selected for validation. Because the read abundance for miR-155-5p in the ipsilateral thalamus was quite low for all samples (TBI: 12.49 (4.61), Sham: 4.56 (0.95), mean (SD)), and there were no commonly predicted targets for miR-146a-5p and miR-155-5p, targets for miR-155-5p were not selected for validation. Quantitative PCR analysis of both targets revealed unaltered gene expression (*p* > 0.05, Figure 5F1,F2).

### 3.5. IsomiRs Identified for the Validated Differentially Expressed miRNAs

**Figure 6:**
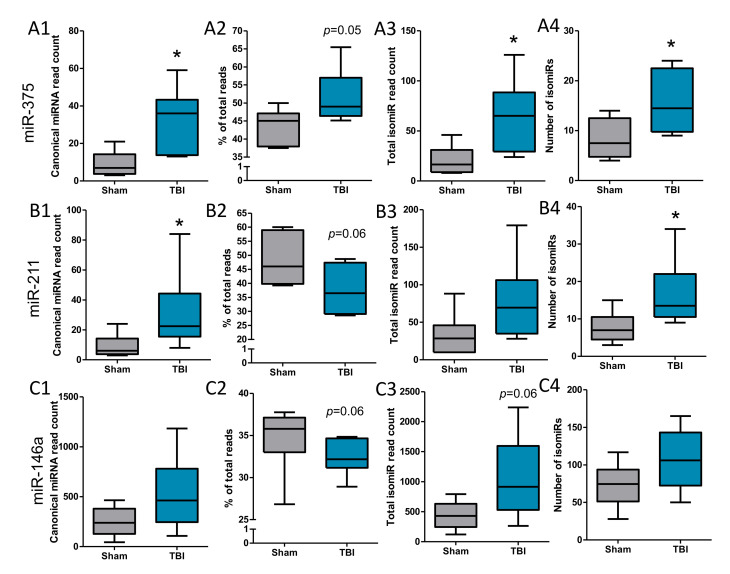
Analysis of canonical (miRbase) miRNA sequence and isomiRs of miR-375, miR-211, and miR-146a. (**A1–A4**) Read count of canonical miR-375 was increased, (**A1**) *p* < 0.05 and there was a trend toward its proportional increase from all miR-375 aligned reads; (**A2**) *p* < 0.1. Additionally, the total isomiR read count (**A3**) *p* < 0.05 and number of isomiRs (**B4**) *p* < 0.05, were increased. ((**B1**–**4**) Similarly, the read count of canonical miR-211 was increased, (**B1**) *p* < 0.05 and there was a trend toward its proportional increase from all miR-211 aligned reads; (**B2**) *p* < 0.1. Total isomiR read count (**B3**) *p* > 0.05 was not changed, but the number of isomiRs (**B4**) *p* < 0.05 was increased. (**C1–C4**) For miR-146a, we observed no significant changes. The trends for canonical miRNA were opposite for cortical differentially expressed miRNAs (miR-375 and miR-211), however, as the proportion of canonical miR-146a was decreased compared to all sequences aligned to it, *p* < 0.1. Statistical significance: *, *p* < 0.05.

To explain the negative miRNA-target expression findings, we performed a detailed analysis of isomiRs. For miR-375-5p, the total number of reads from all isomiRs, and the number of different isomiR types (canonical (Figure 6A1) and isomiRs (Figure 6A3), *p* < 0.05 for both) identified were significantly higher in the TBI animals compared with the sham-operated controls. For miR-211-5p, the number of detected canonical miRNA species was increased (*p* < 0.05, Figure 6B1). The number of different isomiR types identified was significantly higher in the TBI animals compared with the sham-operated controls (Figure 6B3, *p* < 0.05), but the total number of reads from all isomiRs was not significantly different (Figure 6B4, *p* > 0.05). For miR-146a-5p, the total number of reads from all isomiRs, as well as the number of different isomiR types identified, did not differ significantly between the TBI and sham-operated controls (Figure 6C1–C4, *p* > 0.05). For miR-155-5p, no isomiRs were detected. For the four endogenous controls or non-validated differentially expressed miRNA candidates in both the perilesional cortex and ipsilateral thalamus, the total number of reads from all isomiRs and the number of different isomiR types identified did not differ significantly between the TBI and sham-operated controls (*p* > 0.05). The amount of canonical miRNA and isomiRs related to different miRNAs, however, was highly variable.

Next, we performed a detailed analysis of different isomiRs observed for validated differentially expressed miRNAs. For miR-375-3p, the canonical miRNA sequence (TTTGTTCGTTCGGCTCGCGTGA) formed on average 32% of the total read count in the TBI animals (32.42 (2.15)), and 35% in the sham animals (34.68 (3.96)) (*p* = 0.05, Figure 6A2). For miR-211-5p, the canonical sequence (TTCCCTTTGTCATCCTTTGCCT) formed on average 52% of the total read count in the TBI animals (51.6 (7.46)), and 44% in the sham animals (43.64 (4.89)) (*p* = 0.06, Figure 6B2). For miR-146a-5p, the canonical sequence (TGAGAACTGAATTCCATGGGTT) formed on average 38% of the total read count in the TBI animals (37.71 (8.56)), and 48% in the sham animals (48.32 (9.33)) (*p* = 0.06, Figure 6C2).

**Figure 7:**
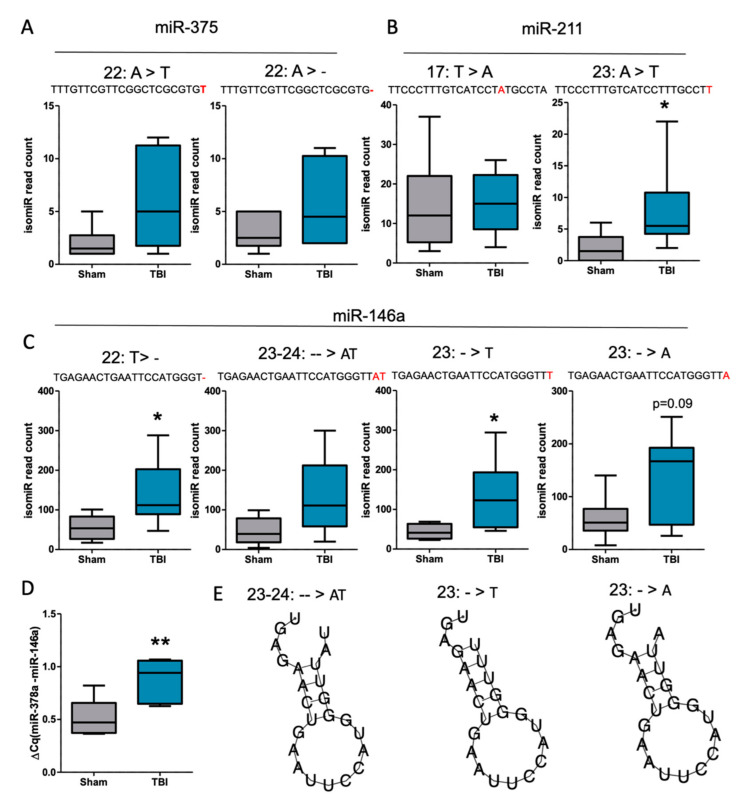
Analysis of isomiR types after traumatic brain injury (TBI). Analysis of cortical differentially expressed miRNAs revealed an increase, especially in 3′ end modifications (**A**,**B**). The miR-211 isomiR 5′–TTCCCTTTGTCATCCTTTGCCTT was significantly increased after TBI (B, *p* < 0.05). For miR-146a, (**C**) several isomiRs were detected. These 2 isomiRs showed increased modifications at the 3′ end (5′– TGAGAACTGAATTCCATGGGT- and 5′– TGAGAACTGAATTCCATGGGTTT; *p* < 0.05). Quantitative RT-PCR chemistry detecting all 3′ end isomiRs (**D**) (FC = 1.7, *p* < 0.05) revealed a difference between controls and post-TBI animals. Primer assay targeting only canonical miR-146a did not detect this increase (see Supplementary Figure S3, FC = 1.4, *p* > 0.05). Interestingly, RNA folding assessment predicted the linear miR-146a form to change to a hairpin-like structure when insertions were detected in the miRNA 3′ end (**E**). Statistical significance: *, *p* < 0.05; **, *p* < 0.01.

Details of the top 2–4 isomiRs (among the top six) for miR-375-3p, miR-211-5p, and miR-146a-5p that were detected from all TBI samples are summarized in Figure 6. Most of these were 3′-isomiRs, i.e., additions, deletions, or nucleotide changes to the canonical miRNA sequence that occurred at the 3′ end. The top two isomiRs detected for miR-375-3p and miR-211-5p were not significantly different between the TBI and controls (*p* > 0.05, Figure 7A,B). For miR-146a-5p, among the top four isomiRs, TGAGAACTGAATTCCATGGGT and TGAGAACTGAATTCCATGGGTTT were significantly more abundant in the TBI animals compared with the controls (*p* < 0.05, Figure 7C). Next, we performed RT-qPCR to detect all 3′ end isomiRs (Figure 7D, FC = 1.7, *p* < 0.05) and were able to detect a difference between the controls and post-TBI animals. Quantitative RT-PCR and primer assay targeting only canonical miR-146a was not able to detect this increase (see Supplementary Figure S3, FC = 1.4, *p* > 0.05). Interestingly, RNA folding assessment predicted that the linear miR-146a form changes to a hairpin-like structure when insertions were detected in the miRNAs 3′ end (Figure 7E).

### 3.6. Elevation of Transfer RNA-Derived Fragments after TBI

**Figure 8:**
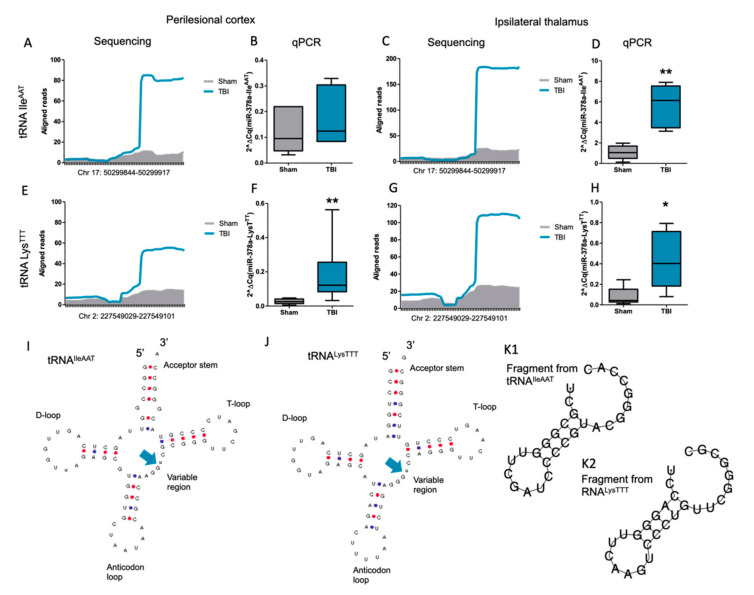
Transfer RNA fragments (tRFs) cleaved from tRNA IleAAT and tRNA LysTTT were upregulated in the perilesional cortex and ipsilateral thalamus after traumatic brain injury (TBI). (**A**) Alignment of the detected reads toward tRNA IleAAT in sham-operated controls (gray shading) and post-TBI rats (blue line) in the perilesional cortex samples. (**B**) Quantitative RT-PCR analysis showed a trend toward upregulation of 3′tRF-IleAAT (FC = 1.5, *p* > 0.05). (**C**) Alignment of the detected reads toward tRNAIleAAT in sham-operated controls (gray shading) and post-TBI rats (blue line) in the ipsilateral thalamus samples. (**D**) Quantitative RT-PCR analysis showed upregulation of 3′tRF-IleAAT (FC = 5.4, *p* < 0.01). (**E**) Alignment of the detected reads toward tRNA LysTTT in sham-operated controls (gray shading) and post-TBI rats (blue line) in the perilesional cortex samples. (**F**) Quantitative RT-PCR analysis showed upregulation of 3′tRF-LysTTT (FC = 6.9, *p* > 0.01). (**G**) Alignment of the detected reads toward tRNALysTTT in sham-operated controls (gray shading) and post-TBI rats (blue line) in the ipsilateral thalamus samples. (**H**) Quantitative RT-PCR analysis showed upregulation of 3′tRF-LysTTT (FC = 5.2, *p* < 0.05). (**I**) Most common cleavage site of fragments from tRNA IleAAT detected based on analysis presented in panels (**A** and **C**). (**J**) Most common cleavage site of fragments from tRNA LysTTT detected based on analysis presented in panels (**E** and **G**). (**K1,K2**) Predicted RNA folding structure for 3′tRFs upregulated after TBI. Statistical significance: *, *p* < 0.05; **, *p* < 0.01.

We observed a clear upregulated profile of tRFs detected by small RNA sequencing in both the perilesional cortex (Supplementary Figure S4A) and ipsilateral thalamus (Supplementary Figure S4B) visualized by unsupervised hierarchical clustering. Using IGV to visualize aligned sequences, we selected two candidate tRNAs, tRNA IleAAT and tRNA LysTTT, for further inspection. Upregulation of fragments cleaved from these two tRNAs was evident in both the perilesional cortex (Figure 8A for tRNA IleAAT and Figure 8E for tRNA LysTTT) and the ipsilateral thalamus (Figure 8C for tRNA IleAAT and Figure 8G for tRNA LysTTT). Interestingly, both tRNAs had a clear cleavage site of tRFs in their variable region [40] producing mainly 27–28 nt long fragments from the 3′ end of the tRNA (Figure 8I for tRNA IleAAT and Figure 8J for tRNA LysTTT). Estimation of a possible secondary structure predicted hairpin-like folding of both fragments (Figure 8K1 for 3′tRF-IleAAT and Figure 8K2 for 3′tRF-LysTTT). Quantitative RT-PCR analysis showed a trend toward the upregulation of fragments originating from tRNA IleAAT in the perilesional cortex (FC = 1.5, *p* > 0.05; Figure 8B). Similar to small RNA-Seq analysis, however, RT-qPCR indicated robust upregulation of the fragments from tRNA IleAAT in the ipsilateral thalamus (FC = 5.4, *p* < 0.01; Figure 8D). Instead, RT-qPCR replicated the observed upregulation of fragments cleaved from tRNA LysTTT in both the perilesional cortex (FC = 6.9, *p* < 0.01; Figure 8F) and the ipsilateral thalamus (FC = 5.2, *p* < 0.05; Figure 8H). We used agarose gel electrophoresis to confirm the size of the detected tRFs, as total RNA (including also pre-tRNA and mature tRNA molecules) was used for the RT-qPCR analysis. Indeed, when only 20–50 nt long RNA was selected after the size separation of cortical RNA samples and subsequent RT-qPCR, a similar upregulated profile of 3′tRF-IleAAT (2.1, *p* > 0.05, *n* = 3 per group) and 3′tRF-LysTTT (3.3, *p* > 0.05, *n* = 3 per group) was detected compared with the total RNA samples (Supplementary Figure S5A for 3′tRF-IleAAT and Supplementary Figure S5C for 3′tRF-LysTTT). Analysis indicated a moderate correlation, meaning that the higher the expression in the total RNA sample, the greater the amount of 3′tRF also detected after the size separation (Supplementary Figure S5B for 3′tRF-IleAAT (Spearman r = 0.54, *p* > 0.05) and Supplementary Figure S5D for 3′tRF-LysTTT (Spearman r = 0.75, *p* > 0.05).

### 3.7. mRNA Targets Predicted for the Validated tRF Candidates

**Figure 9:**
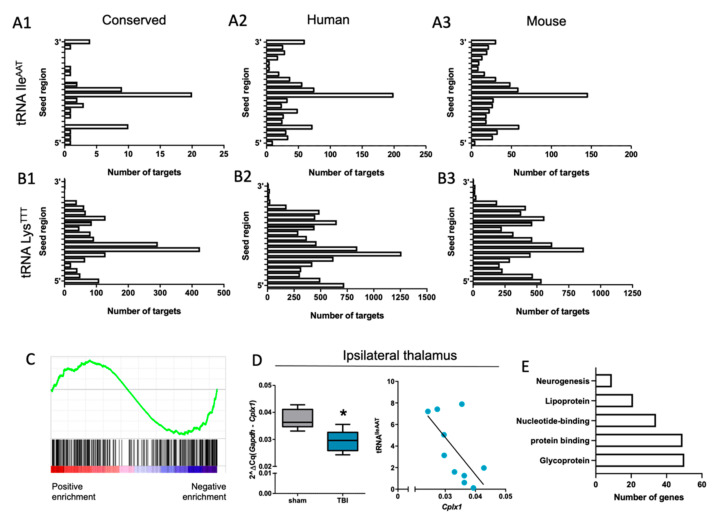
Downregulation of the predicted targets of upregulated tRFs after traumatic brain injury. (**A1–A3**) Number of predicted targets of 3′tRF-IleAAT for conserved, human and mouse 3′-untranslated regions (UTRs) of protein coding genes. (**B1–B3**) Number of predicted targets of 3′tRF-LysTTT for conserved, human, and mouse 3′-UTRs of protein-coding genes. (**C**) Gene set enrichment analysis of predicted targets of both tRFs (combined gene list) in the ipsilateral thalamus revealed negative enrichment (ES = 0.22, FDR <0.01). (**D**) Quantitative RT-PCR analysis validated downregulation of one selected target candidate of 3′tRF-IleAAT, Cplx1 (FC = 0.78, *p* < 0.05). Further, the lower the Cplx1 expression level, the greater the detection of 3′tRF-IleAAT in the same sample (Spearman r = 0.43, *p* < 0.05). (**E**) Functional annotation of negatively enriched predicted tRF targets linked them to glycoprotein, protein binding, nucleotide-binding, lipoprotein, and neurogenesis (*p* < 0.05, DAVID functional annotation tool). Statistical significance: *, *p* < 0.05.

We used an 8-nt approach [39] to predict mRNA targets for validated 3′ tRFs (Figure 9A1–A3 for 3′tRF-IleAAT and Figure 9B1–B3 for 3′tRF-LysTTT). Gene set enrichment analysis indicated a negative enrichment of these predicted targets for a combined target list of both tRFs in the ipsilateral thalamus (ES = −0.22, FDR <0.01, Figure 9C). The thalamus was selected for further investigation as both fragments were robustly upregulated in this brain area. We selected one candidate target (Cplx1) of 3′tRF-IleAAT for further validation with RT-qPCR. In the ipsilateral thalamus, Cplx1 was downregulated (FC = 0.78, *p* < 0.05, Figure 9D) and the lower the expression level, the greater the amount of 3′tRF-IleAAT detected in the same sample (Spearman r = 0.43, *p* < 0.05, Figure 9D). Gene expression of Cplx1 showed a trend toward downregulation in the perilesional cortex (FC = 0.4, *p* > 0.05; Spearman r = 0.38, *p* > 0.05; *n* = 5 per group; Supplementary Figure S6) where the upregulation of tRF was also not that high. Further, functional annotation of negatively enriched predicted tRF targets linked them to glycoprotein, protein binding, nucleotide-binding, lipoprotein, and neurogenesis (*p* < 0.05, Figure 9E, Supplementary Spreadsheet 1).

### 3.8. No Clear Upregulation of tRF-Cleaving Enzyme Angiogenin after TBI

RNAseq experiments revealed that the tRF-cleaving enzyme angiogenin was upregulated in the ipsilateral thalamus (log2FC = 0.47, FDR <0.01, Supplementary Figure S7A). We quantified the expression level of angiogenin with RT-qPCR in both the perilesional cortex (FC = 1.26, *p* > 0.05) and ipsilateral thalamus (FC = 1.62, *p* > 0.05, n = 6 for controls and *n* = 5 for TBI) but did not detect the expected significant upregulation (Supplementary Figure S7B). A moderate correlation, however, was observed between angiogenin expression and the 3′tRF levels (Spearman r = 0.45, *p* < 0.05 for 3′tRF-IleAAT and Spearman r = 0.36, *p* < 0.05 for 3′tRF-LysTTT) (Supplementary Figure S7C for 3′tRF-IleAAT and Supplementary Figure S7D for 3′tRF-LysTTT) in the thalamus.

### 3.9. Elevated 3′tRF and miR-146a Levels Relate to Worse Behavioral Outcome after TBI

**Figure 10:**
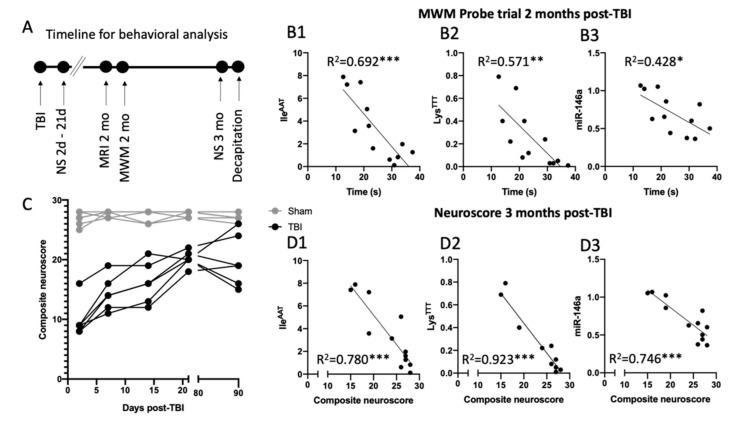
Upregulation of tRFs in the ipsilateral thalamus correlated with a worse behavioral outcome after traumatic brain injury (TBI). (**A**) Neuromotor function was assessed by the composite neuroscore at acute and chronic time-points post-TBI, and memory deficits were investigated with the Morris water maze at 2 months post-TBI. (**B1–B3**) Correlation analysis showed that rats showing deficits in the probe trial, represented by less time spent in the correct quadrant, also had the highest expression of the full miR-146a profile (all isomiRs included; Spearman r = 0.43, *p* < 0.05, panel (**B1**)), 3′tRF-IleAAT (Spearman r = 0.70, *p* < 0.001; panel (**B2**)), and 3′tRF-LysTTT (Spearman r = 0.57, *p* < 0.01; panel (**B3**)). (**C**) TBI rats exhibited acute motor function deficits compared with controls at 2 days post-TBI (*p* < 0.01) with recovery to subacute at 7 days (*p* < 0.01, compared with controls), 14 days (*p* < 0.01, compared with controls) and 21 days (*p* < 0.01, compared with controls) post-TBI time-points. At 3 months post-TBI, high variance in the measured score was observed between rats, but lower scores were still observed compared with controls (*p* < 0.01). (**D1**–**D3**) Correlation analysis indicated a relation between a lower neuroscore value at 3 months post-TBI and increased expression of the full miR-146a profile (all isomiRs included; Spearman r = 0.75, *p* < 0.001; panel (**D1**)), 3′tRF-IleAAT (Spearman r = 0.78, *p* < 0.001, panel (**D2**)) and 3′tRF-LysTTT (Spearman r = 0.92, *p* < 0.001; panel (**D3**)). Statistical significance: *, *p* < 0.05; **, *p* < 0.01; ***, *p* < 0.001.

We assessed neuromotor functions with the composite neuroscore at acute and chronic time-points post-TBI and investigated memory deficits in the Morris water maze at 2 months post-TBI (Figure 10A). Our correlation analysis showed that rats exhibiting deficits in the probe trial, represented by less time spent in the correct quadrant, also had the highest expression of the full miR-146a profile (all isomiRs included; Spearman r = 0.43, *p* < 0.05, Figure 10B1), 3′tRF-IleAAT (Spearman r = 0.70, *p* < 0.001; Figure 10B2) and 3′tRF-LysTTT (Spearman r = 0.57, *p* < 0.01; Figure 10B3). As expected, all TBI rats exhibited acute motor deficits compared with the controls at 2 days post-TBI (*p* < 0.01, Figure 10C) with recovery toward subacute at the 7 days (*p* < 0.01, Figure 10C), 14 days (*p* < 0.01, Figure 10C) and 21 days (*p* < 0.01, Figure 10C) post-TBI time-points. When the neuroscore was assessed at 3 months post-TBI, the measured score varied widely between rats. The difference between post-TBI rats and the controls remained significant (*p* < 0.01, Figure 10C). Interestingly, our correlation analysis indicated a relation between the lower neuroscore value at 3 months post-TBI and the increased expression of the full miR-146a profile (all isomiRs included; Spearman r = 0.75, *p* < 0.001; Figure 10(D1)), 3′tRF-IleAAT (Spearman r = 0.78, *p* < 0.001, Figure 10(D2)) and 3′tRF-LysTTT (Spearman r = 0.92, *p* < 0.001; Figure 10D3).

## 4. Discussion

A growing body of evidence demonstrates the involvement of different non-coding RNAs in brain diseases, including TBI [41,42,43,44]. MicroRNAs have been identified as key regulators of inflammation after brain injuries [45,46]. In the present study, our objective was to identify chronically altered miRNA and tRF signatures after TBI. Our data indicate that two upregulated miRNAs (miR-155 and miR-146a) in the thalamus at a chronic time post-TBI might have unfavorable effects for recovery after TBI. The inflammatory role of the dysregulated miRNAs in the perilesional cortex, however, is not clear. Further, we revealed a dysregulated profile of isomiRs of quantified differentially expressed miRNAs, and a novel class of small RNAs, the tRFs, involved in post-TBI pathophysiology. For the first time, we report here a relation between upregulated tRFs and an unfavorable behavioral outcome after TBI.

### 4.1. Chronic Neuroinflammation after TBI

Brain inflammation is a prominent feature after TBI that has long-lasting secondary injury mechanisms and evolves in the brain for months after TBI [47,48]. The control of neuroinflammation may lessen the risk of developing comorbidities, like memory deficits and epilepsy [47,49]. Yet, brain inflammation is sparsely studied at chronic time-points post-TBI. Importantly, many inflammatory mediators remain dysregulated at 3 months post-TBI, as demonstrated by our previous genome-wide transcriptomic analysis [4]. Here we demonstrated a network level dysregulation of protein-coding genes in the perilesional cortex and ipsilateral thalamus. As expected, we observed robust regulation of inflammation-related pathways and regulators in both brain areas. Our analysis also highlighted the regulation of genes involved in organismal injury and abnormalities, and cell and tissue morphology. Perilesional cortex-specific changes were observed in axonal guidance signaling pathways. Further, ipsilateral thalamus-specific changes were observed in functions of molecular transport and cell death, as well as toxic effects of mitochondrial dysfunction. Interestingly, our analysis revealed broad regulation of the pathways and functions connected to hematologic system development and function. Early coagulopathies have been reported in TBI patients and even in patients with isolated severe TBI without severe bleeding [50,51,52]. Importantly, trauma-induced coagulopathies are independent predictors of poor outcomes and are linked to secondary injuries. How regulation of the hematologic system in the brain influences secondary injuries and relates to chronic neuroinflammation remains to be studied.

### 4.2. Differentially Expressed miRNAs after TBI

We detected relatively few changes in canonical miRNAs in both brain areas investigated. Based on earlier studies, the neuroinflammatory role of the upregulated miRNAs (miR-375 and miR-211) in the perilesional cortex is not well known. Upregulation of miR-375, however, is considered to protect the brain from ischemic stroke resulting in a reduced infarct volume and decreased cell apoptosis [53]. On the other hand, knockdown of human miR-375 upregulates NF-κB and pro-inflammatory factors, such as tumor necrosis factor-α, IL-1β, IL-6 and IL-8, in the colorectal cancer cell line Caco-2 [54]. These findings suggest the possible anti-inflammatory and neuroprotective roles of miR-375. Further, miR-211 excess regulation is connected to perturbation of learning functions in the Morris water maze [55]; mice with high miR-211 levels lost the capacity to locate the platform quadrant in a probe trial assessing reference memory. Interestingly, in our study, post-TBI rats also showed decreased ability to find the platform in the Morris water maze test and had higher cortical miR-211 levels. On the other hand, higher levels of miR-211 seem to protect the brain from hypersynchronization, and nonconvulsive and convulsive seizures [55]. These findings suggest multileveled, beneficial and harmful, roles of miR-211 in the brain.

MiR-146a is upregulated in the brains of post-TBI patients, animal models of TBI, and similarly in epilepsy patients and animal models of epilepsy [56] and miR-146a is suggested to be involved in the development and progression of seizures, especially through the regulation of inflammation and immune responses [57,58]. A recent study [59] indicated that the silencing of miRNA-146a decreases oxidative stress and the inflammatory response in a rat model of temporal lobe epilepsy (TLE); one of the genes involved in the process was Notch1. This is interesting as we previously reported upregulation of Notch1 in the dentate gyrus at 3 months post-TBI [60]. In this study, Notch1 was upregulated in the perilesional cortex (log2FC = 0.46, FDR <0.01); however, in the ipsilateral thalamus—the brain area where miR-146a was upregulated—we observed no significant change (Log2FC = 0.26, FDR >0.05). Notch1 did not come up in our miRNA-target analysis as we were interested in significantly changed targets. It is still possible that miR-146a downregulates the protein level of Notch1 and decreases its mRNA level in the thalamus. Our earlier study showed an increase in miR-155 in the rat and human brain post-TBI [56]. This upregulation was associated with activated glial cells. In vitro studies indicate that human astrocytes acquire a pro-inflammatory phenotype and overexpress miR-155 after stimulation with a pro-inflammatory macrophage-conditioned culture medium. Thus, miR-155-promoted neuroinflammation via astrocyte activation is involved in the secondary injury. Moreover, numerous studies have demonstrated a pro-inflammatory role of miR-155 [46,61].

### 4.3. Are 3′isomiRs a Specific Feature of TBI?

General information on the unique mature sequence of each miRNA is cataloged in the miRBase database [62]. These listed sequences are called miRNA canonical forms. The sncRNAseq experiments, however, suggest that miRNA lengths and sequences could be modified in animal and human tissues. These RNA isoforms are called isomiRs [63]. Experiments demonstrate substantial overlap in functional mRNA networks suppressed by both canonical miRNAs and their isomiRs, and that these isomiRs are functionally relevant [63].

Detailed analyses of sncRNAseq data have identified especially high fractions of mature miRNAs containing one or more 3′ nucleotide additions [16,64]. These 3′ nucleotide additions most commonly include single and double nucleotide additions of adenine and/or uridine bases. Studies investigating these modifications have found that only a small fraction of miRNAs derived from a particular miRNA locus are subject to, for example, 3′ adenine addition (0–20%). These additions could therefore provide a mechanism for the cell to regulate the targeting efficiency of the transcripts derived from specific miRNA and this profile has been shown to be cell type-specific [16]. Functional distinction between isomiR production between vertebrates and Drosophila [16] emphasizes that miRNA modifications may have divergent roles across groups of animals and humans. For some evolutionarily conserved miRNA families, however, generally comparable rates of additions have been observed across species [16,65]. Overall, very diverse and contradictory functions are attributed to these addition events [17]. These functions include miRNA targeting efficiency, exosome localization, degradation, stabilization, and influencing associations with argonaute proteins.

In this study we especially observed 3′ nucleotide additions or modifications. Further, our prediction of RNA folding suggested possible alterations in the folding structure of miR-146a. Moreover, we were unable to detect any changes in the predicted targets of the canonical miRNAs analyzed. Whether or not 3′ nucleotide isomiRs are involved in post-TBI pathology and how each detected nucleotide modification/addition affects miRNA functionality in the brain remains to be studied.

### 4.4. Variable Region Cleaved 3′tRFs Are Upregulated after TBI

Transfer RNAs and tRFs support different cellular processes, from canonical functions in translation to recently unraveled mechanisms as gene expression regulators [24,66]. Interestingly, the recently described TRF-AGO2 association leads to target repression that is similar to miRNA activity, thereby acting on posttranscriptional regulation [66]. In this study, we reported an increase in 3′tRFs. Reports suggest that this particular tRF class regulates gene expression in a miRNA-like manner [67]. Indeed, we found numerous predicted target candidates for these dysregulated tRFs and showed decreased expression of Cplx1. Functional annotation of negatively enriched predicted tRF targets linked them to glycoprotein, protein binding, nucleotide-binding, lipoprotein, and neurogenesis.

As noted, Cplx1 is predicted to be one of the affected targets of 3′tRF-IleAAT. Cplx1 belongs to the complexin/synaphin gene family and positively regulates exocytosis of various cytoplasmic vesicles, such as synaptic vesicles and other secretory vesicles [68]. This protein organizes the soluble NSF attachment protein receptor proteins (SNAREs). Thus, it can prevent SNAREs from releasing neurotransmitters until an action potential arrives at the synapse [68]. Interestingly, a study by Yi et al. [69] reported transient increases in the levels of Cplx1 and Cplx2 proteins in the ipsilateral cortex 6 h following lateral FPI. This increase was followed by a decrease in Cplx1 in the ipsilateral cortex and hippocampus, and a decrease in both complexins in the ipsilateral thalamus at day 3 and day 7 post-injury. The authors suggested that alterations in complexin levels may play an important role in neuronal cell loss following TBI, and thus contribute to the post-TBI pathophysiology. We have now expanded this finding and report chronic downregulation of Cplx1 gene expression in the ipsilateral thalamus still at 3 months post-TBI and its possible regulation through 3′tRF-IleAAT.

Variable region-cleaved tRFs are rarely described in the literature [70] and their biogenesis is not well known; however, tRF cleaving is generally mediated by endoribonucleases, such as angiogenin and dicer [20]. Angiogenin is a component of the acute-phase response that protects the organism from microbial and environmental stress [71]. Knockdown of angiogenin sensitizes cells to stress and promotes stress-induced activation of apoptotic caspases [72]. Treatment of cultured mammalian cells with angiogenin stimulates tRNA cleavage [73]. Research related to amyotrophic lateral sclerosis revealed that angiogenin plays an important role in the endogenous protective pathways of motor neurons exposed to hypoxia [74]. Other studies reported that angiogenin is secreted from motor neurons and taken up by astroglia where it induces tRNA cleavage [75] that targets specific mRNAs together with argonaute proteins modulating cell survival following stress [19]. We found no clear indication of the regulatory role of angiogenin, however, as a single explanatory factor for variable region-cleaved tRFs. We observed no change in the level of dicer (data not shown), another tRF cleaving endonuclease. A recent study suggested that the human RNase T2 generates long and short tRFs in vitro [76]. Interestingly, Rnaset2 was robustly upregulated in our mRNA-Seq analysis in both the perilesional cortex (Log2FC = 0.74, FDR <0.01) and ipsilateral thalamus (Log2FC = 1.39, FDR <0.01) after TBI. Detailed functional in vitro studies are needed to unveil the role of RNase T2 in variable region-cleaved tRF production.

Lastly, our data suggest that increases in the 3′tRFs strongly correlate with miR-146a (Supplementary Figure S8), a miRNA previously linked to reactive astrocytes [77]. Whether 3′tRF-IleAAT and 3′tRF-LysTTT are indeed increased in reactive astrocytes chronically after TBI or whether they co-exist with this pathologic feature remains to be studied. Further, it is not yet known how elevated tRF levels after TBI affect mature tRNA. Whether the overall role of tRFs is pro-inflammatory or anti-inflammatory remains to be explored. Despite a single study demonstrating increased seizure susceptibility and dysfunctional neuronal transmission as a result of mutated tRNA n-Tr20 [26], the link between tRNA and epileptic-associated phenotypes remains unclear. Moreover, Hogg et al., described tRFs that were modulated in an in vitro seizure model and could predict seizure imminence in human epilepsy patients when measured from plasma [25]. Finally, the present study demonstrated a link between a worse behavioral outcome after TBI and increased 3′tRF levels in the perilesional cortex and ipsilateral thalamus.

## 5. Conclusions

These results suggest a possible functional role of 3′tRF-IleAAT and 3′tRF-LysTTT after TBI and emphasize the importance of future studies to elucidate their mechanisms of action in chronic neuroinflammation and worse recovery outcomes after TBI. Moreover, additional studies are needed to reveal the importance and roles of isomiRs in disease mechanisms and functional outcome.

## Data Availability

All RNA-Seq raw data were saved to the NCBI Gene Expression Omnibus (GEO; series accession number GSE192979 (mRNAseq) and GSE192980 (small RNAseq).

## Supplementary Materials

The following are available online at https://www.mdpi.com/article/10.3390/biomedicines10010136/s1. Methods: Quantitative RT-PCR analysis of canonical miR-146a; Figures S1: Number of upregulated (A) and downregulated (B) genes in the perilesional cortex and ipsilateral thalamus; Figures S2: Mapping of the small non-coding RNA sequencing reads; Figures S3: Quantitative RT-PCR analysis of miR-146a did not reveal significant upregulation (FC = 1.4, *p* > 0.05); Figures S4: Unsupervised hierarchical clustering of differentially expressed transfer RNA fragments; Figures S5: Agarose gel electrophoresis to separate 20–50 nucleotide (nt) long small RNA from the total RNA pool; Figures S6: Gene expression of Cplx1 showed a trend toward downregulation in the perilesional cortex (FC = 0.4, *p* > 0.05); Figures S7: Expression of angiogenin (ANG) after experimental traumatic brain injury; Figures S8: Correlation between miR-146a, miR-155, and deregulated transfer RNA-derived fragments; Table S1: RIN integrity numbers prior the sequencing service and later PCR study; Table S2: Quality control of messenger RNA sequencing; Spreadsheet 1.
