# Supplementary material for: Transfer RNA-Derived Fragments and isomiRs Are Novel Components of Chronic TBI-Induced Neuropathology
Source: Biomedicines. 2022 Jan 8;10(1):136. doi: 10.3390/biomedicines10010136 (PMC8773447; doi:10.3390/biomedicines10010136)
Supplement: Supplementary file 1 [file biomedicines-10-00136-s001.zip › Supplementary Material S1.pdf]

Supplementary Data

Supplementary methods

Quantitative RT-PCR analysis of canonical miR-146a

Total RNA was translated to complimentary DNA (cDNA) with a TaqMan miRNA Reverse Transcriptase Kit (#4366596, Applied Biosystems, Foster City, CA, http://www.appliedbiosystems.com), according to the manufacturer's instructions. RT primer for miR-146a-5p (002163, Applied Biosystems) was used. RT was performed using a T100™ Thermal Cycler (Bio-Rad Laboratories Inc, Hercules, CA, USA) as follows: 16°C for 30 min, 42°C for 30 min, 85°C for 5 min, and 4°C thereafter. U6 snRNA (snRNA U6, 001973, Applied Biosystems) was used as an endogenous control. RT-qPCR using the TaqMan Small RNA Assays protocol (Applied Biosystems, http://www.appliedbiosystems.com) was used. Briefly, TaqMan Small RNA Assay (20x, miR-146a-5p (002163, GAGAACTGAATTCCATGGGTT, Applied Biosystems, or snRNA U6, 001973, GTGCTCGCTTCGGCAGCACATATACTAAAATTGGAACGATACAGAGAAGATTAGCATGGCCCCTGCGCAAGGATGACACGCAAATTCGTGAAGCGTTCCATATTTT, Applied Biosystems) was mixed with 1.33 µl of RT product, TaqMan Universal PCR Master Mix II with no UNG, and nuclease-free water (Ambion, AM9938, Thermo Fisher Scientific, Waltham, MA, USA) according to manufacturer's instructions. RT-qPCR was run using the StepOnePlus™ Real-Time PCR System (Software v2.1, Applied Biosystems) with a standard program: 50°C for 2 min, 95°C for 10 min, followed by 40 cycles (15 s each) at 95°C, and finally, at 60°C for 60 s. Every sample was run in triplicate. No template water control was added to the run. Ct values were normalized relative to snRNA U6 with the formula 2-ΔCt [1].

1. Livak, K. J. & Schmittgen, T. D. Analysis of Relative Gene Expression Data Using Real-Time Quantitative PCR and the 2−ΔΔCT Method. *Methods* **25**, 402–408 (2001).

Supplementary Figures

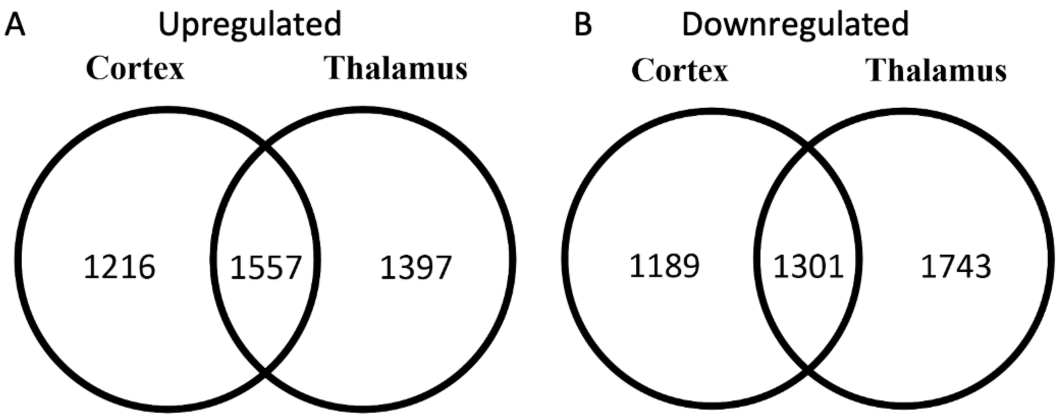

Supplementary Figure S1. Number of upregulated (A) and downregulated (B) genes in the perilesional cortex and ipsilateral thalamus.

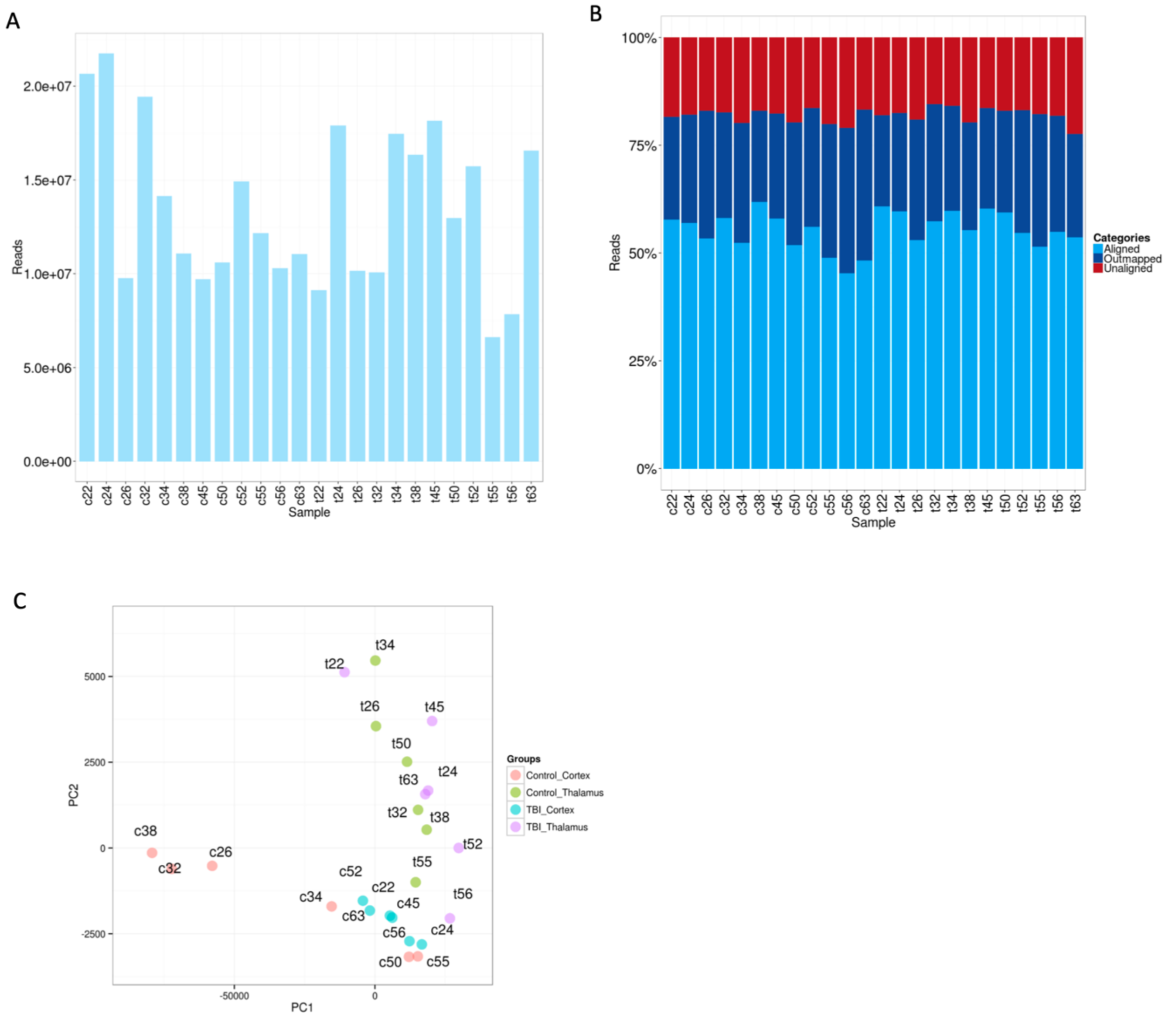

Supplementary Figure S2. Mapping of the small non-coding RNA sequencing reads. (A) On average, 13.5 million reads were obtained per sample. The figure below shows the total number of reads per sample. (B) The plot shows that the fraction of mappable reads (=counts) is within the normal range and the distribution is similar for all samples. A typical microRNA sequencing experiment yields approximately 40%–60% microRNAs mapping to the reference genome. (C) Principal component analysis (PCA) plot. The PCA was performed on all using top 50 microRNAs with the highest coefficient of variation (based on TPM normalized reads).

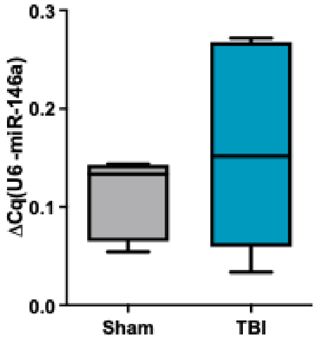

Supplementary Figure S3. Quantitative RT-PCR analysis of miR-146a did not reveal significant upregulation (FC=1.4, p>0.05).

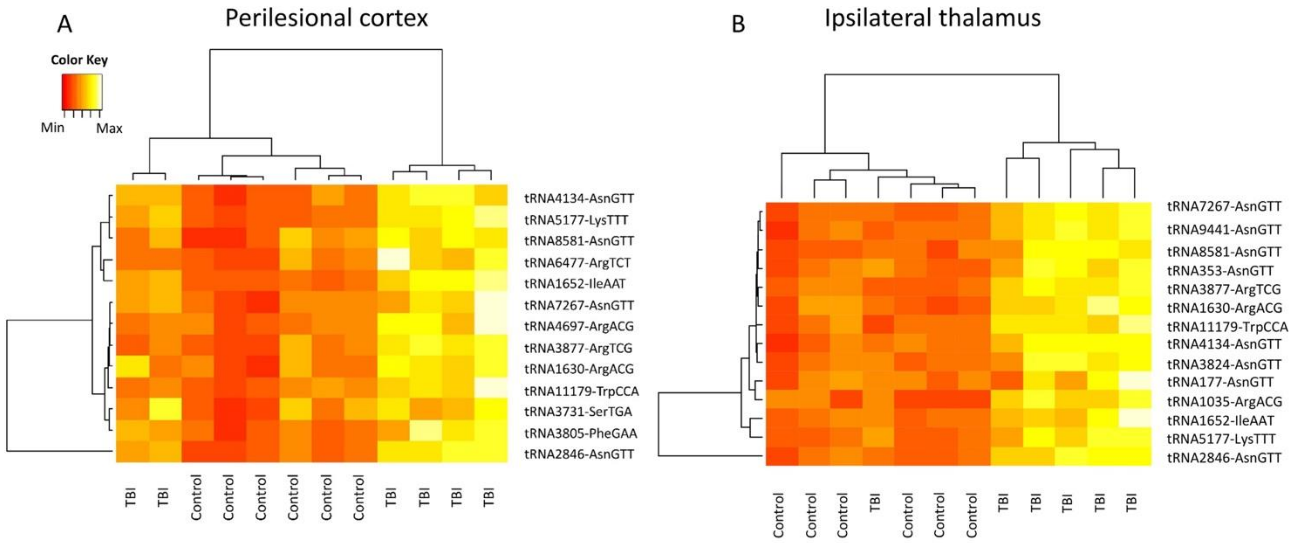

Supplementary Figure S4. Unsupervised hierarchical clustering of differentially expressed transfer RNA fragments (FDR>0.05) after traumatic brain injury in the perilesional cortex (A) and ipsilateral thalamus (B). Abbreviations: Asn, asparagine; Arg, arginine; Ile, isoleucine; Lys, lysine; Phe, phenylalanine; Ser, serine; Trp, tryptophan.

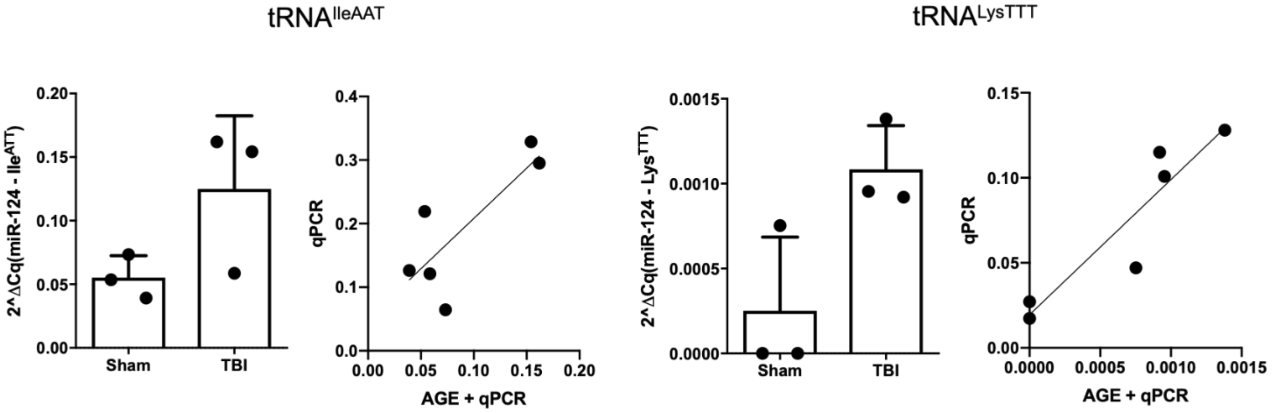

Supplementary Figure S5. Agarose gel electrophoresis to separate 20–50 nucleotide (nt) long small RNA from the total RNA pool. (A) When only 20–50nt long RNA was selected after the size separation of cortical RNA samples and before RT-qPCR, an upregulated profile of 3'tRF-Ile^{AAT} (2.1, $p>0.05$, $n=3$ per group) was observed. (B) Analysis indicated a moderate correlation, meaning that the higher the expression in the total RNA sample, the greater the amount of 3'tRF detected after the size separation (Spearman $r=0.54$, $p>0.05$). (C) Similar analysis showed more 3'tRF-Lys^{TTT} (3.3, $p>0.05$, $n=3$ per group) in post-TBI samples compared with sham-operated controls. (D) Analysis indicated moderate correlation, meaning that the higher the expression in the total RNA sample, the greater the amount of 3'tRF detected after the size separation (Spearman $r=0.75$, $p>0.05$).

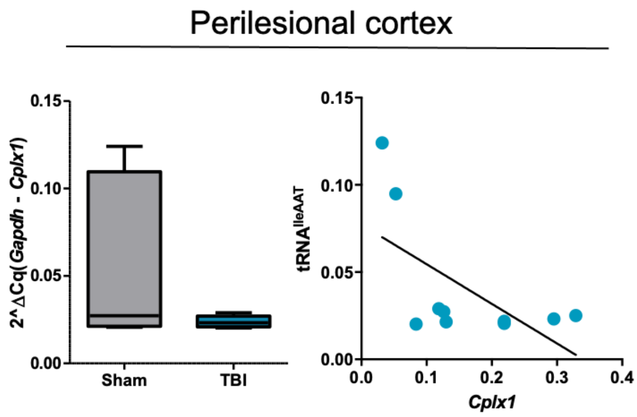

Supplementary Figure S6. Gene expression of *Cplx1* showed a trend toward downregulation in the perilesional cortex (FC=0.4, $p>0.05$). No clear correlation was observed between 3'tRF-Ile^{AAT} and *Cplx1* (Spearman $r=0.38$, $p>0.05$; $n=5$ per group).

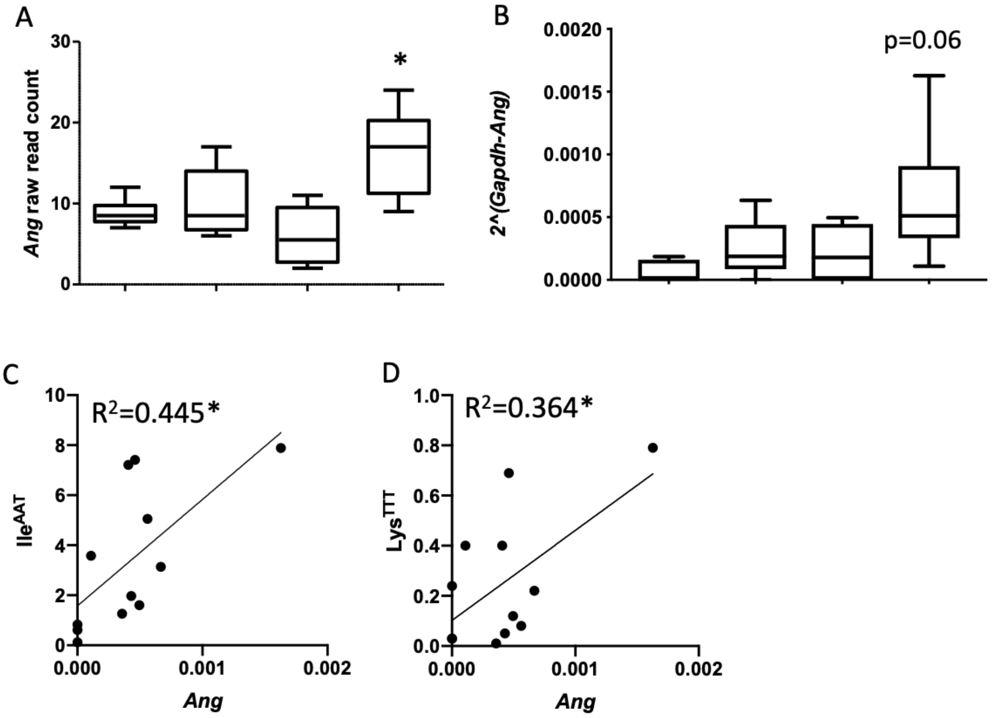

Supplementary Figure S7. Expression of angiogenin (ANG) after experimental traumatic brain injury. (A) ANG was upregulated in the ipsilateral thalamus (Log2FC=0.47, FDR<0.01) but not in the perilesional cortex (FDR>0.05). (B) Quantitative RT-PCR showed a trend toward upregulation in both the perilesional cortex (FC=1.26, p>0.05) and ipsilateral thalamus (FC=1.62, p<0.1), but the effect was not statistically significant. (C-D) A mild correlation between ANG and 3'tRFs (Spearman r=0.45, p<0.05 for 3'tRF-Ile^{AAT} and Spearman r=0.36, p<0.05 for 3'tRF-Lys^{TTT}) expression levels, however, was observed (panel C 3'tRF-Ile^{AAT} and panel D for 3'tRF-Lys^{TTT}) in the ipsilateral thalamus.

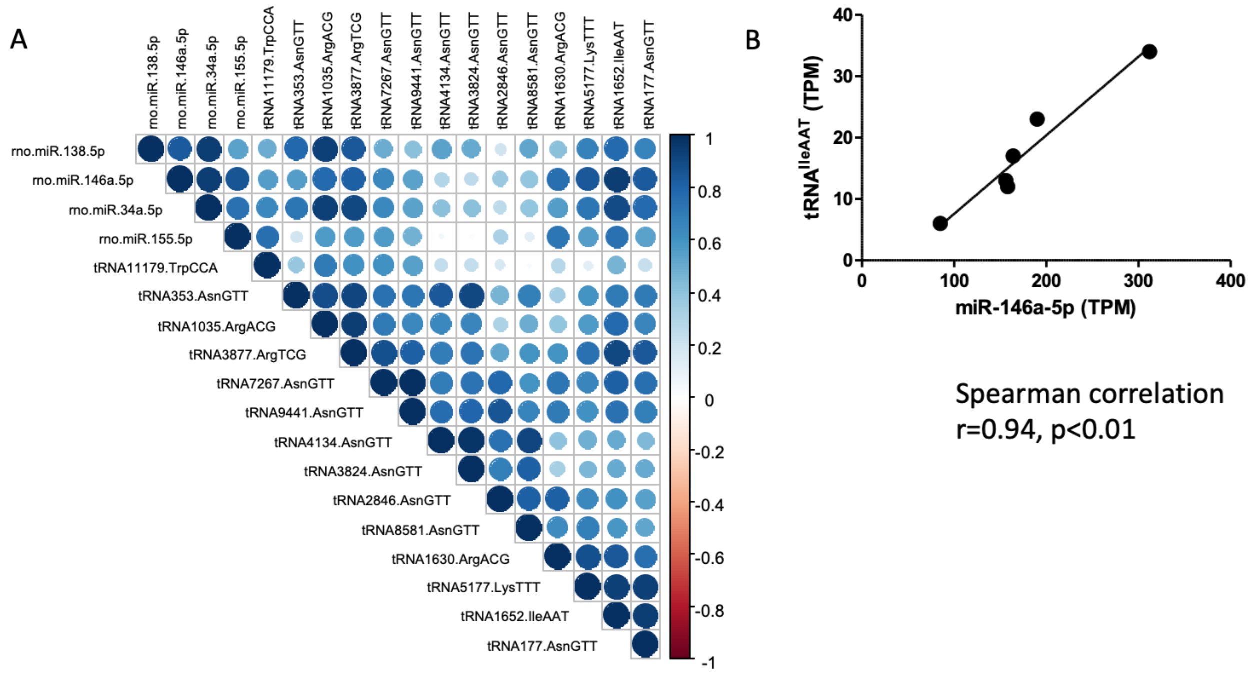

Supplementary Figure S8. Correlation between miR-146a, miR-155, and deregulated transfer RNA-derived fragments. (A) Correlation matrix reveals associations between small non-coding RNAs in the ipsilateral thalamus after traumatic brain injury. (B) Higher miR-146a indicates higher 3'tRF-Ile^{AAT} (Spearman correlation r=0.94, p<0.01). Abbreviations: Abbreviations: Asn, asparagine; Arg, arginine; Ile, isoleucine; Lys, lysine; Trp, tryptophan.

Supplementary Tables

Supplementary Table S1. RIN integrity numbers prior the sequencing service and later PCR study.

	NANODROP 260/280	AGILENT RIN
THA22	2.05	9.00
CX22	2.06	8.90
THA24	2.05	9.20
CX24	2.04	9.10
THA45	2.10	9.00
CX45	2.07	9.10
THAA52	2.10	9.10
CX52	2.10	9.20
THA56	2.07	9.00
CX56	2.08	9.10
THA63	2.03	9.10
CX63	2.08	9.00
THA26	2,.9	9.00
CX26	2.07	9.00
THA32	2.01	9.20
CX32	2.03	9.00
THA34	2.05	9.20
CX34	2.06	9.00
THA38	2.04	9.10
CX38	2.07	9.00
THA50	2.07	9.10
CX50	2.07	9.00
THA55	2.07	9.10
CX55	2.07	9.10

THA, thalamus
Cx, cortex

Supplementary Table S2. Quality control of messenger RNA sequencing.

		Outmapped reads			
Sample	Total reads	rRNAs (%)	Other (mtRNA) (%)	Mapped reads (%)	Unmappable reads (%)
t38	52064186	0.064	11.623	86.356	1.943
c50	47305296	0.11	12.34	85.695	1.838
t45	46447521	0.117	8.741	89.307	1.823
c45	57014336	0.237	9.052	88.796	1.905
c22	60806193	0.496	10.627	86.997	1.866
c63	42628454	0.135	10.468	87.416	1.966
t50	49646919	0.115	10.662	87.425	1.784
t22	47893172	0.154	9.354	88.674	1.81
c32	61980326	0.093	11.418	86.441	2.035
t52	51412123	0.328	9.279	88.483	1.898
t26	53182734	0.062	10.052	88.073	1.797
t55	52517099	0.125	9.667	88.302	1.894
t34	40716080	0.126	10.649	87.423	1.784
t63	43744534	0.129	10.225	87.743	1.882
c55	45937472	0.26	10.515	87.329	1.873
c24	57106046	0.627	7.158	90.263	1.936
t56	69906025	0.142	10.523	87.455	1.876
t24	39066640	0.078	9.313	88.782	1.812
t32	48955220	0.087	12.896	85.262	1.74
c38	45535090	0.119	11.764	86.257	1.85
c56	51794020	0.13	10.338	87.726	1.79
c52	60831605	0.316	10.519	87.277	1.872
c34	55755093	0.426	11.969	85.754	1.839
c26	48718370	0.248	11.713	86.271	1.761
